# Transcriptomic Analysis of STAT1/3 in the Goat Endometrium During Embryo Implantation

**DOI:** 10.3389/fvets.2021.757759

**Published:** 2021-10-14

**Authors:** Haokun Liu, Caixia Wang, Zuhui Li, Chunmei Shang, Xinyan Zhang, Ruixue Zhang, Aihua Wang, Yaping Jin, Pengfei Lin

**Affiliations:** ^1^College of Veterinary Medicine, Northwest A&F University, Xianyang, China; ^2^Key Laboratory of Animal Biotechnology, Ministry of Agriculture and Rural Affairs, Northwest A&F University, Xianyang, China

**Keywords:** goat, interferon tau, embryo implantation, RNA-Seq, STAT1, STAT3

## Abstract

Interferon tau (IFNT), a pregnancy recognition signal in ruminants, promotes the establishment of embryo implantation by inducing the expression of interferon-stimulated genes (ISGs) via the Janus kinase/signal transducer and activator of transcription (JAK/STAT) signaling pathway. However, the precise regulatory mechanism of IFNT in goat embryo implantation remains largely unknown. In this study, we performed RNA sequencing of goat endometrial epithelial cells (gEECs) with or without 20 ng/mL IFNT treatment. Differential comparison showed that there were 442 upregulated differentially expressed genes (DEGs) and 510 downregulated DEGs. Bioinformatic analyses revealed that DEGs were significantly enriched in immune-related functions or pathways. The qRT-PCR validation results showed that the expression levels of STAT family members (STAT1, STAT2, and STAT3) were significantly upregulated in gEECs after IFNT treatment, which is in agreement with the RNA-seq data. Meanwhile, the protein levels of p-STAT1 and p-STAT3 increased significantly in gEECs after 6 and 24 h of IFNT treatment, respectively. Further *in vivo* experiments also confirmed that both mRNA and protein phosphorylation levels of STAT1 and STAT3 in the uterus on day 18 of pregnancy (P18) were significantly increased compared to those on day 5 (P5) and day 15 of pregnancy (P15). On P5, STAT1 and STAT3 proteins were primarily located in the uterine luminal epithelium (LE) and glandular epithelium (GE), and were also detected in the stromal cells. The intense immunostaining of STAT1 and STAT3 proteins were decreased on P15 and then increased on P18, especially in the superficial GE and subepithelial stromal cells. Moreover, p-STAT1 and p-STAT3 were highly expressed in the deep GE on P18. Collectively, these results highlight the role of IFNT in regulating endometrial receptivity in gEECs and uncover the temporal and spatial changes in the expression of STAT1/3 during embryo implantation in the goat endometrium.

## Introduction

Embryo implantation is crucial for successful pregnancy. During this period, the elongated conceptus and receptive endometrium recognize each other and have complex interactions, which change the expression of a number of genes, resulting in promotion of embryo implantation and placenta formation, eventually leading to the establishment of pregnancy (1, 2). Implantation failure is the major cause of pregnancy loss in cattle, accounting for 30%–50% of all cases (3, 4). Although many studies have been conducted to identify the gene network in the endometrium or conceptus during embryo implantation (5–8), the precise molecular mechanism has not been well-characterized.

Interferon tau (IFNT) is a type I IFN secreted from trophectoderm cells of the ruminant conceptus, which is instrumental in the pregnancy recognition of ruminants in the synchronization of the conceptus and maternal uterus (9). In addition to its anti-luteolytic action, IFNT regulates the function of the receptive endometrium and the elongation of the conceptus to facilitate the establishment of pregnancy (10). Upon binding to the receptor, IFNT performs these functions through the Janus kinase/signal transducer and activator of transcription (JAK/STAT) signaling pathway. In the JAK/STAT signal transduction process, JAK1 and Tyrosine kinase 2 (Tyk2) successively phosphorylate STAT1 and STAT2 (11, 12). The phosphorylated-STAT1/STAT2 heterodimer forms the interferon-stimulated gene factor 3 (ISGF3) complex with interferon regulatory factor 9 (IRF9) and translocates to the nucleus, which binds to IFN-stimulated response elements (ISREs) in the promoter region of a group of interferon-stimulated genes (ISGs), leading to the transcription of ISGs (13). In addition, type I IFN induces STAT3 phosphorylation (14). Similar to other STATs, STAT3 forms complexes with other transcription factors, including the STAT3 homodimer (15) and STAT1/STAT3 heterodimer (16), and translocates to the nucleus for signal transduction.

Notably, the mRNA levels of critical signaling components of the JAK/STAT pathway (STAT1, STAT2, and IRF9) are low in the endometrial luminal epithelium (LE) during the peri-implantation period in sheep (17). In cattle, nuclear STAT1 expression is reduced in the LE during the peri-implantation period when compared with that during the pre-implantation period, indicating a decrease in phosphorylated STAT1 protein levels and subsequent inhibition of ISGF3 synthesis (18). However, a detailed analysis of the expression profile of STATs in the goat uterus during embryo implantation has not been reported. Additionally, although the JAK/STAT3 signaling pathway is one of the key pathways in decidualization (19, 20), its role in ruminants remains unknown.

Therefore, to explore the potential function of JAK/STAT3 during early pregnancy in goats, based on the gene regulatory network of IFNT-treated goat endometrial epithelial cells (gEECs) by RNA sequencing, we determined the expression and localization of STATs in the goat endometrium during the peri-implantation period.

## Materials and Methods

### Tissue Collection

Mature Guanzhong dairy goats (n = 9, aged 2–3 years, average weight = 59.28±1.93 kg) were reared in the experimental animal center of Northwest A & F University, Yangling, China. The goats exhibiting at least two estrous cycles of normal duration were used in this study. At estrus, female goats were mated with fertile males to induce natural pregnancy, which was recorded as day 0 of pregnancy. Pregnancy was confirmed on day 5 (P5, in the pre-implantation period) by recovering blastocysts from the uterus. Pregnancy at day 15 (P15, in the pregnancy recognition period) and day 18 (P18, in the embryo adhesion period) was identified by observing the elongated tubular conceptus and linear conceptus in the uterus, respectively. The uteri of pregnant goats on P5 (n = 3), P15 (n = 3), and P18 (n = 3) were collected immediately after the goats were subjected to midventral laparotomy and hysterectomy. The uterine tissues were fixed in 4% (v/v) paraformaldehyde in phosphate-buffered saline (PBS) without Ca^2+^/Mg^2+^ (PBS) or immediately frozen in liquid nitrogen.

### Cell Culture and Treatment

Immortalized gEECs were obtained as previously described (21). The gEECs were cultured in Dulbecco's minimum essential medium nutrient mixture F-12 (DMEM/F-12, BasalMedia, China) supplemented with 10% fetal bovine serum (FBS, ZETA LIFE, USA) at 37°C in a humidified atmosphere of 5% CO_2_. When gEECs reached 50% confluence, the cells treated with or without 20 ng/mL recombinant ovine IFNT (Sangon Biotech) for 6 h were used for RNA-seq analysis. Meanwhile, gEECs treated with or without 20 ng/mL IFNT for 6, 12, and 24 h were used for qRT-PCR confirmation.

### RNA Isolation and Quality Assessment

Total RNA of gEECs or uterus tissue was isolated using RNAiso Plus (Takara, Japan), and genomic DNA was removed using the *Evo M-MLV* RT Kit (Accurate Biology, China) according to the manufacturer's protocols. The purity, concentration, and integrity of the total RNA were checked using a NanoPhotometer® spectrophotometer (IMPLEN, CA, USA), Qubit® RNA Assay Kit in Qubit® 2.0 Fluorometer (Life Technologies, CA, USA), and RNA Nano 6000 Assay Kit of the Bioanalyzer 2,100 system (Agilent Technologies, CA, USA).

### RNA Sequencing

Library preparation, clustering, and sequencing for RNA sequencing were performed according to the manufacturer's recommendations. Briefly, ribosomal RNA was removed from 3 μg of total RNA using the Epicenter Ribo-zero™ rRNA Removal Kit (Epicenter, USA). Subsequently, RNA-seq libraries were generated using the Library Prep Kit for Illumina® (NEB, USA). The library fragments were purified using the AMPure XP system (Beckman Coulter, Beverly, USA). Next, 3 μL of USER Enzyme (NEB, USA) was used with adaptor-ligated cDNA before PCR. PCR was performed with Phusion High-Fidelity DNA polymerase, Universal PCR primers, and Index (X) Primer. The products were then purified using the AMPure XP system and library quality was assessed using the Agilent Bioanalyzer 2,100 system. Clustering was performed on a cBot Cluster Generation System using the TruSeq PE Cluster Kit v3-cBot-HS (Illumina). After cluster generation, the RNA-seq libraries were sequenced on an Illumina HiSeq 4,000 platform. The sequencing data generated in this study were deposited in the NCBI GEO database (https://www.ncbi.nlm.nih.gov/geo/query/acc.cgi?acc=GSE184110).

### RNA-seq Data Analysis

Raw reads were aligned to the goat genome using HISA T2 (v2.0.4) software. Reference genome and gene model annotation files were downloaded directly from the genome website. Transcriptome assembly was performed using StringTie (v1.3.1) software in a reference-based approach. Gene expression abundance was assessed by calculating the FPKMs of genes in each sample using Cufflinks (v2.1.1) software. Differentially expressed gene analysis was performed using the Ballgown suite. Transcripts with an adjusted *p*-value <0.05 were assigned as “differentially expressed.” *P*-values were adjusted using the Benjamini–Hochberg procedure.

### GO and KEGG Enrichment Analyses

Gene Ontology (GO) enrichment analysis of differentially expressed genes (DEGs) was performed using the GOseq R package. KEGG enrichment analysis of DEGs was performed using the KOBAS software. GO terms and KEGG with *q*-values <0.05 were considered significantly enriched by DEGs.

### qRT-PCR

Reverse transcription was performed using the *Evo M-MLV* RT Kit (with gDNA Eraser, Accurate Biology, China) according to the manufacturer's protocol. PCR was performed using the ChamQ SYBR qPCR Master Mix (Vazyme, China). Data collection and analysis were performed on a CFX Connect machine (Bio-Rad, USA) using CFX Manager software. All quantitative PCRs were performed in triplicate. Primers were synthesized by Tsingke Biotechnology Co., Ltd. (China), and their sequences are listed in Supplementary Table 1. Relative mRNA expression levels of target genes were normalized against the relative quantity of GAPDH mRNA in the same sample, and calculated by the formula using the 2^−ΔΔCt^ method.

### Western Blotting

The gEECs and uterus tissues were lysed using a protein extraction kit (KeyGEN BioTECH, China) to obtain total protein. After 10 min of lysis at 4°C, the samples were centrifuged at 12,000 g at 4°C for 10 min to collect the supernatant for concentration determination. The concentration of protein samples was determined using a BCA kit (KeyGEN BioTECH, China). Next, the protein samples were added to loading buffer (5×, CWBIO, China) and heated at 100°C for 10 min using a DryBath (Thermo Fisher Scientific, USA). For western blotting, the protein samples were separated by SDS-polyacrylamide gel electrophoresis (SDS-PAGE) on gels and blocked with 5% non-fat milk (BBI, China) in Tris-buffered saline Tween (TBST) for 1.5 h at 25°C after being transferred to a PVDF membrane (ZETA LIFE, USA). Following blocking, the membranes were incubated with anti-STAT1 (1: 2,000, diluted in TBST, Cellular Signaling Technology, USA), anti-Phospho-STAT1 (Tyr701, 1: 2,000, Cellular Signaling Technology, USA), anti-STAT3 (1: 2,000, Cellular Signaling Technology, USA), anti-Phospho-STAT3 (Tyr705, 1: 2,000, Cellular Signaling Technology, USA), and anti-β-actin (1: 2,000, Sanjian Biotech, China) specific primary antibodies at 4°C overnight and horseradish peroxidase (HRP) -conjugated secondary antibodies (1: 8,000, diluted in TBST) at 25°C for 2 h. After incubation, the membranes were washed with TBST. Then, G: BOX Chemi XRQ (Syngene, USA) was used to capture images and analyze the relative intensity of the protein.

### Immunohistochemistry

The uterine tissues were fixed for 24 h, dehydrated through a graded ethanol series, and embedded in paraffin. Next, 5-μm-thick sections were mounted onto glass slides pre-coated with poly L-lysine solution (Sigma, USA) and incubated overnight at 37°C. After deparaffinization and rehydration, sections were placed in citrate buffer (pH 6.0). Antigen retrieval was performed by heating the sections for 20 min in a microwave oven at 95°C, and the slides were washed in PBS. The sections were then stained with the streptavidin-peroxidase method using an UltraSensitiveTM SP (Rabbit or Mouse) IHC Kit (Maixin Biotechnologies, China). Briefly, the sections were pretreated with 0.3% (v/v) H_2_O_2_ for 40 min at 37°C to quench endogenous peroxidase activity. After washing with PBS, the sections were incubated with 10% pre-immune serum for 70 min at 37°C. After blocking, the sections were incubated with anti-STAT1 (1: 800, diluted in PBS), anti-Phospho-STAT1 (Tyr701, 1: 100), anti-STAT3 (1: 300), and anti-Phospho-STAT3 (Tyr705, 1: 100) at 4°C overnight, and rewarmed at 37°C for 40 min, washed with PBS, and incubated with biotin-labeled goat anti-rabbit or anti-mouse IgG at 25°C for 40 min. The sections were then washed with PBS and incubated with streptavidin-biotin peroxidase for 40 min at 25°C. Thereafter, the sections were visualized with diaminobenzidine (DAB), lightly counterstained with hematoxylin for 25 s, dehydrated, and coverslipped. As a negative control, the primary antibody was substituted with pre-immune serum. Sections were imaged under a microscope (Nikon, Germany) after drying at 25°C.

### Statistical Analysis

Data are presented as the mean ± standard error of the mean (SEM). Statistical significance was set at *p* < 0.05. All data were representative of at least three different experiments and statistically analyzed using SPSS software (SPSS 20.0, IBM Corp., Armonk, NY, USA). Student's *t*-test was used for comparisons between the two groups. Statistical differences among different groups (>2) were evaluated using one-way ANOVA with multiple comparisons among groups tested by Tukey's *post hoc* test.

## Results

### Differentially Expressed Genes Analysis

**Figure 1:**
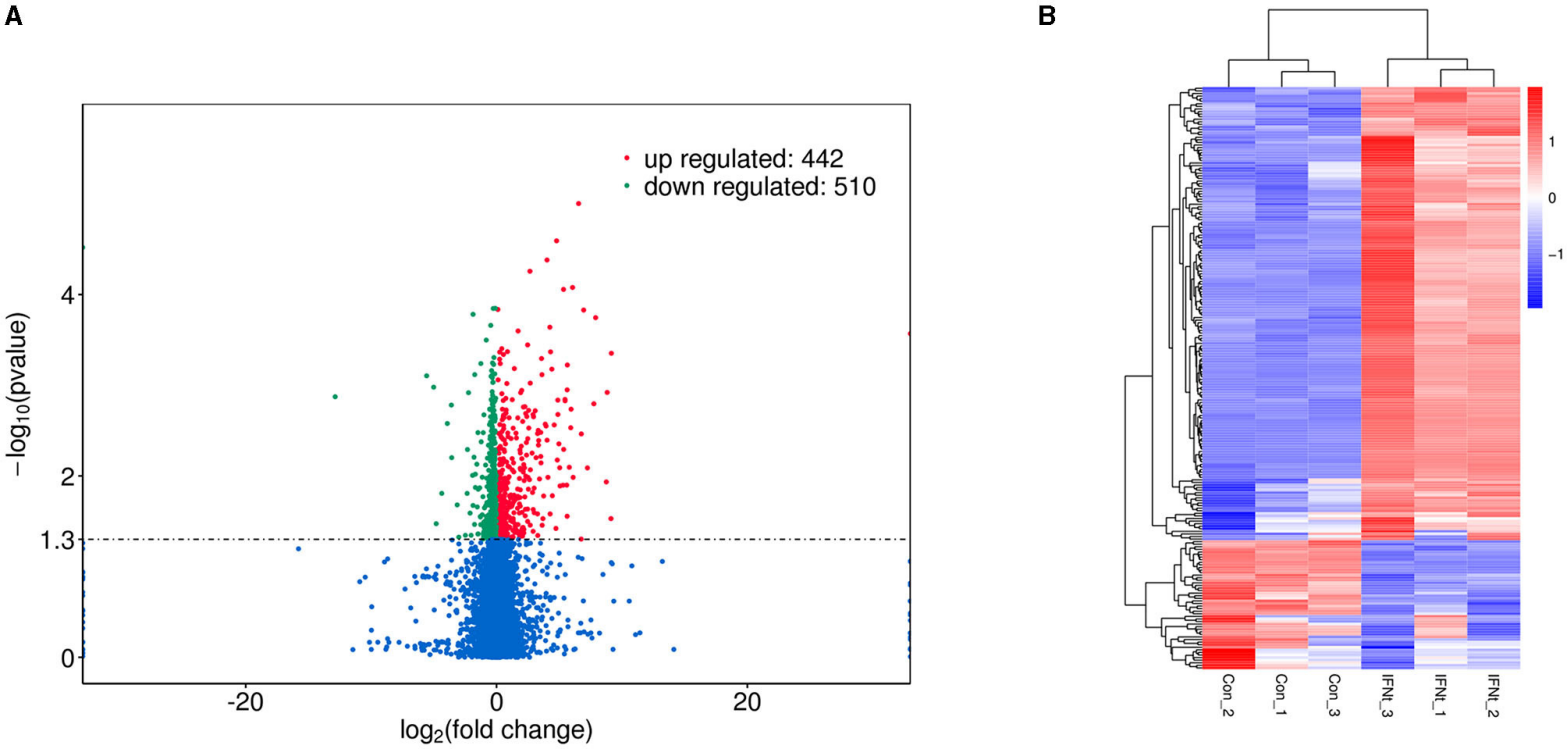
Volcano plot and heatmap of DEGs in IFNT-treated gEECs. (**A)** Volcano plot. Genes were plotted in green for downregulation and red for upregulation, and non-significant genes are shown as blue points. **(B)** Heatmap. Log_10_ (FPKM + 1) was used for clustering, red for high expression genes, and blue for low expression genes.

We generated triplicate libraries from IFNT-treated and untreated gEECs (control group) and sequenced them to a depth of 89.46 Gb clean data. Alignment of the clean data against the reference genome yielded 82.79–89.78% of the total mapped reads, >90.70% of the Q30, and exceeding 46.38% of the GC content. Multiple mapped reads (3.88–5.46%) were excluded from further analyses. Therefore, 43,779 mRNA transcripts were detected in the 6 samples, indicating that the RNA sequencing results were reliable. After the quantification of gene expression levels, a volcano plot was used to provide an overview of the DEGs (Figure 1A). A total of 952 genes were differentially expressed between the IFNT and control groups. Among these genes, 442 genes were upregulated and 510 were downregulated (Supplementary Table 2). A heatmap depicting the DEGs is shown in Figure 1B.

### GO and KEGG Enrichment Analyses

**Figure 2:**
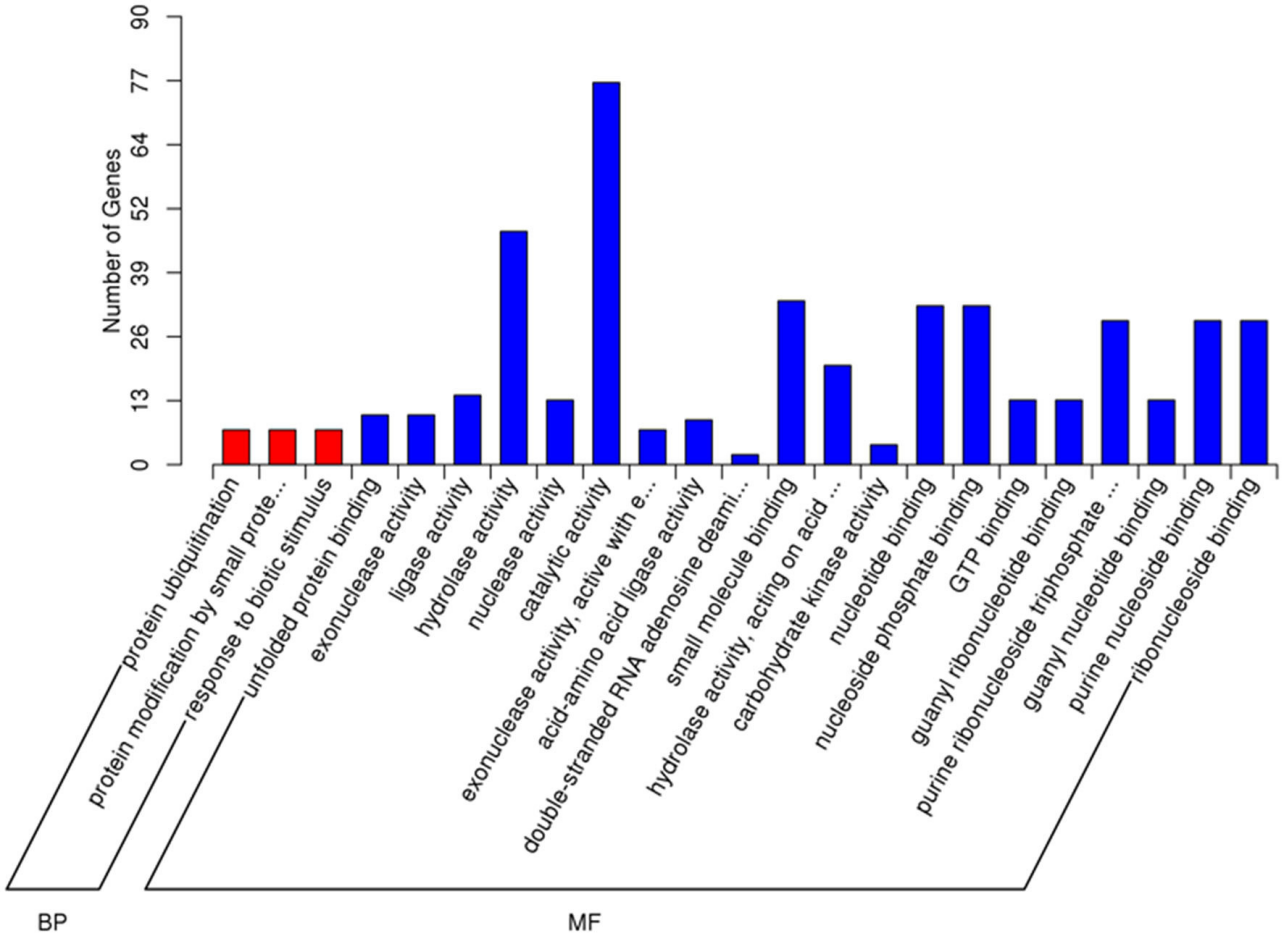
Gene Ontology (GO) enrichment analysis of DEGs in the IFNT-treated gEECs. GO terms with adj. *p*-value <0.05 were considered significantly enriched by DEGs. BP, biological process; MF, molecular function.

GO and KEGG enrichment analyses were performed to identify the potential biological processes of the DEGs. In the GO analysis performed among IFNT and control groups, the number of upregulated DEGs annotated to GO terms was identified (downregulated DEGs annotated to a few GO terms), and the significantly enriched GO term was covered. The top 23 enriched functions, including three biological processes and 20 molecular functions (Figure 2). Protein ubiquitination (GO: 0016567), protein modification by small protein conjugation (GO: 0032446), and response to biotic stimulus (GO: 0009607) were the primary categories in biological processes. Hydrolase activity (GO: 0016787) and small molecule binding (GO: 0036094) were the main function categories.

**Figure 3:**
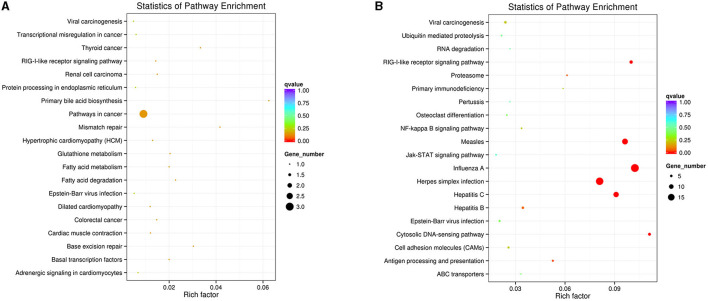
KEGG enrichment analysis of DEGs in the IFNT-treated gEECs. **(A)** KEGG pathway enrichment analysis of downregulated DEGs. **(B)** KEGG pathway enrichment analysis of upregulated DEGs. KOBAS software was used to test the statistical enrichment of DEGs in KEGG pathways. *P*-values were adjusted using Benjamini–Hochberg procedure (adj. *p*-value <0.05).

The DEGs pathway analysis was based on the KEGG pathway database for calculation of the abundant pathways. The top 20 enriched pathways of downregulated and upregulated DEGs are shown in Figures 3A,B. Among the downregulated DEG enrichment pathways, pathways in cancer showed the highest degree of enrichment. Among the upregulated DEG-enriched pathways, the main enriched pathways were those related to influenza A, herpes simplex infection, measles, and hepatitis C.

### STATs Expression in gEECs by IFNT Treatment

**Figure 4:**
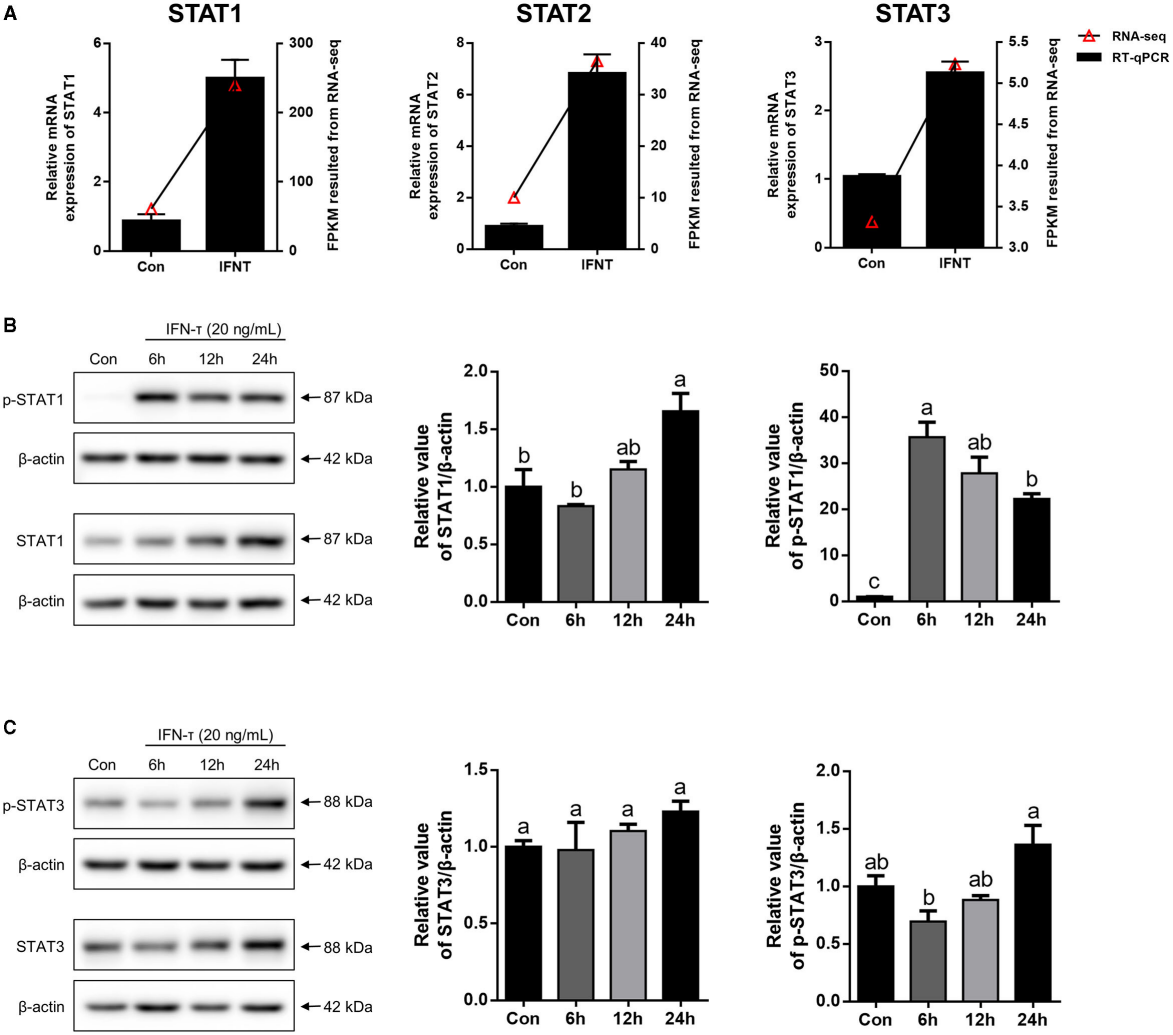
The expression analysis of STATs in IFNT-treated gEECs. **(A)** To validate RNA-seq data, the mRNA level of STAT1, STAT2, and STAT3, which was detected by qRT-PCR after gEECs were treated with 20 ng/mL IFNT for 6 h. Gene expression was normalized to GAPDH. Values represent as the mean ± S.E.M. from three independent experiments, and compared with the control group. (**B,C)** The protein expression of STAT1, p-STAT1, STAT3, and p-STAT3 was analyzed using Western Blotting after 20 ng/mL IFNT treatment for 6, 12, and 24 h. The data are presented as the mean ± S.E.M. from three independent experiments, and bars with different letters (a/b/c) are significantly different (*p* < 0.05).

In agreement with the RNA-seq data, the expression levels of STAT1, STAT2, and STAT3 mRNA were significantly upregulated by IFNT in gEECs measured using qRT-PCR (Figure 4A; *p* < 0.05). Moreover, STAT1 protein levels in gEECs gradually increased with increasing time of IFNT treatment (Figure 3B). Meanwhile, p-STAT1 expression was markedly increased by IFNT treatment and peaked at 6 h (Figure 3B; *p* < 0.05). Although STAT3 protein levels in gEECs were not significantly altered within 24 h of IFNT treatment, p-STAT3 levels were significantly increased after 24 h of IFNT treatment (Figure 4C; *p* < 0.05). Unfortunately, the STAT2 and p-STAT2 antibodies which are suitable for goat tissue, were not detected in this study.

### Expression Analysis of STATs in the Goat Uterus During Embryo Implantation

**Figure 5:**
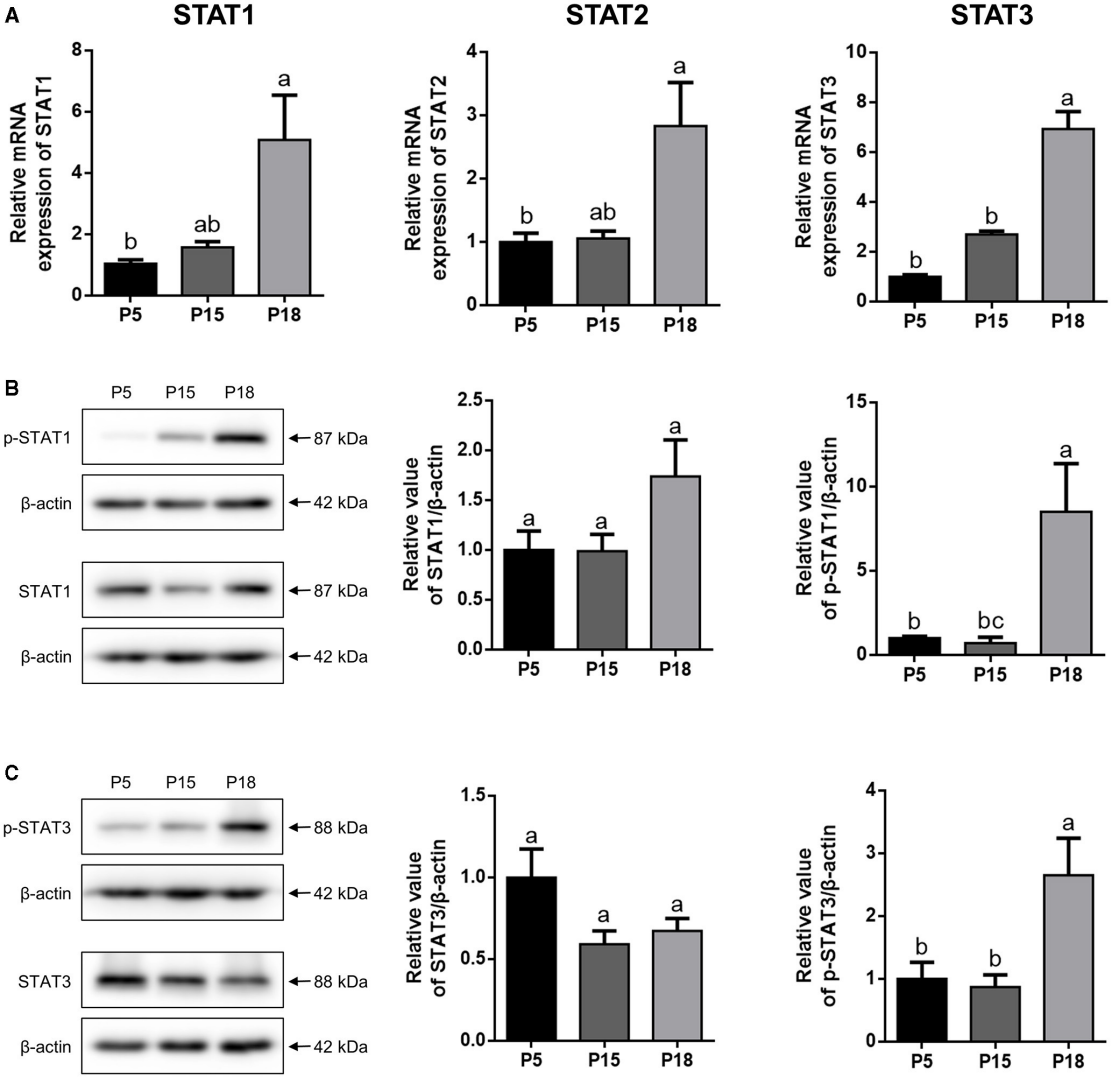
The expression analysis of STATs in goat uterus during early pregnancy. **(A)** Relative mRNA level of STAT1, STAT2, and STAT3 was detected by qRT-PCR in pregnant goat uterus on post-mating days 5 (P5), 15 (P15), and 18 (P18). Gene expression was normalized to GAPDH. **(B,C)** The protein expression of p-STAT1, STAT1, p-STAT3, and STAT3 in goat uterus was analyzed by Western Blotting. All the data are presented as the mean ± S.E.M. from three independent experiments, and bars with different letters (a/b/c) are significantly different (*p* < 0.05).

As shown in Figure 5A, the mRNA levels of STAT1, STAT2, and STAT3 in the uterus were higher on P18 than on P5 and P15, respectively (*p* < 0.05). However, there was no significant difference in STAT1, STAT2, and STAT3 mRNA expression levels between P5 and P15. Furthermore, no obvious changes in the STAT1 and STAT3 proteins were detected among P5, P15, and P18, whereas p-STAT1 and p-STAT3 were markedly upregulated on P18 (Figures 5B,C; *p* < 0.05).

### Localization of STAT1/3 in the Goat Uterus During Embryo Implantation

**Figure 6:**
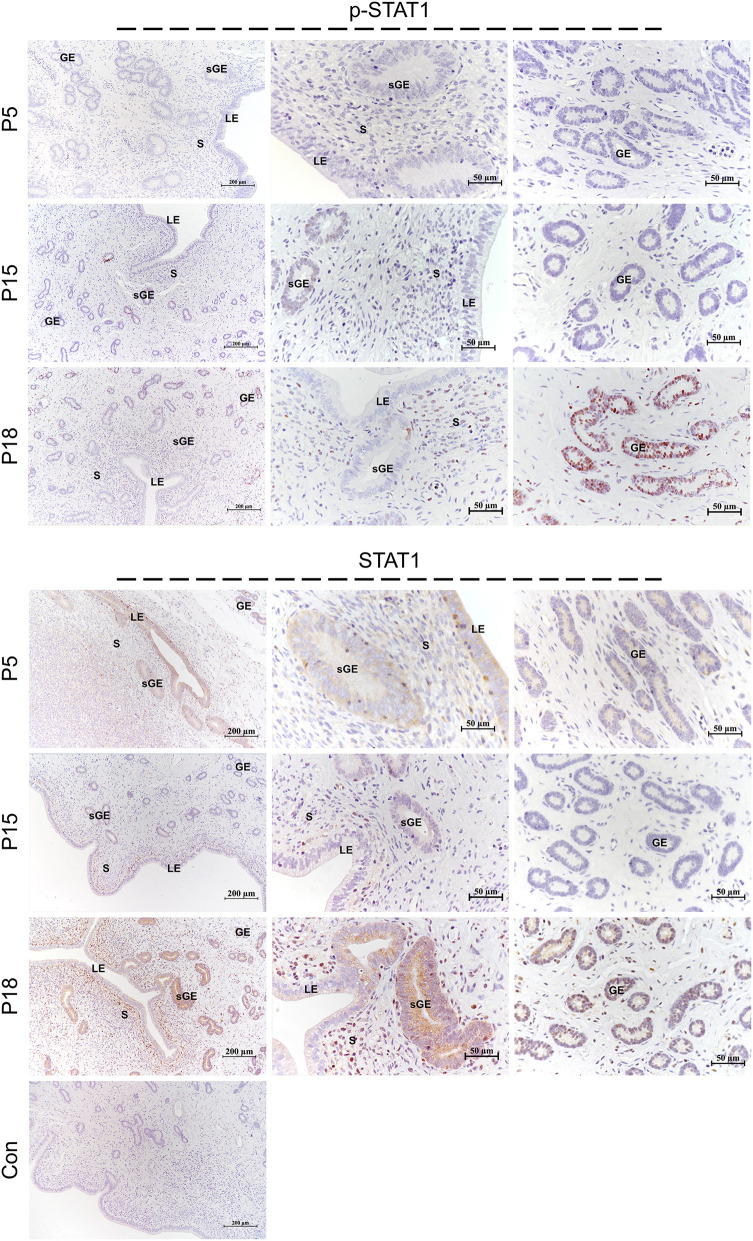
Immunolocalization of p-STAT1 and STAT1 in goat uterus during early pregnancy. For Control, preimmune serum was substituted for the primary antibody. LE, luminal epithelium; GE, glandular epithelium; sGE, superficial glandular epithelium; S, stroma.

**Figure 7:**
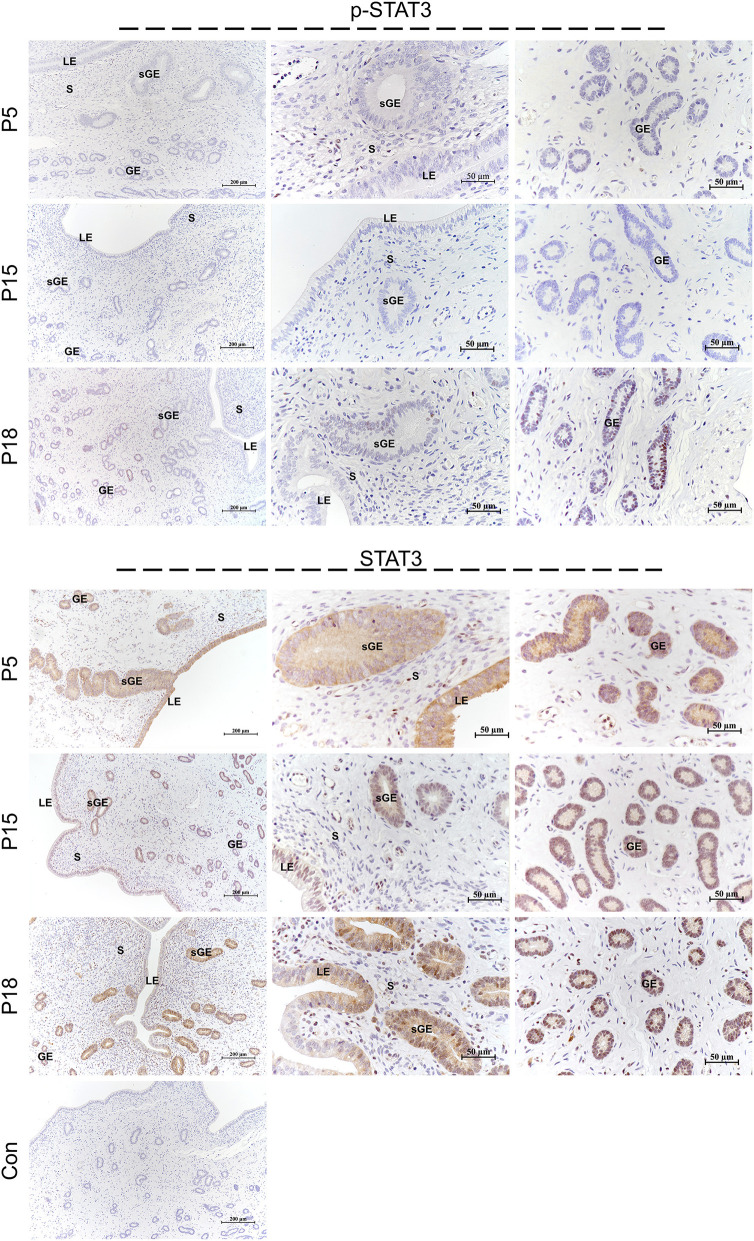
Immunolocalization of p-STAT3 and STAT3 in goat uterus during early pregnancy. For Control, preimmune serum was substituted for the primary antibody. LE, luminal epithelium; GE, glandular epithelium; sGE, superficial glandular epithelium; S, stroma.

STAT1 protein was mainly localized in the LE and glandular epithelium (GE) on P5, and slight immunostaining of the STAT1 protein was detected in the stromal cells (Figure 6). On P15, weak STAT1 immunostaining was only weakly observed in superficial GE (sGE) and subepithelial stromal cells. STAT1 expression was significantly increased in endometrial cells and was particularly abundant in the GE and subepithelial stromal cells on P18. Meanwhile, no obvious immunostaining of p-STAT1 protein was detected in the endometrium on P5. The p-STAT1 protein was slightly expressed in the sGE on P15; the expression gradually increased and was strongly detected in the deeper GE on P18. The STAT3 protein was widely distributed in the endometrial cells on P5, P15, and P18 (Figure 7). Moreover, p-STAT3 was only expressed in the GE at P18 (Figure 7).

## Discussion

In this study, we first provide a relatively comprehensive DEG profile for gEECs. Based on the RNA-seq results, we found that the expression and localization of STAT1 and STAT3, which are downstream components of the JAK-STAT signaling pathway, showed spatiotemporal changes during embryo implantation process.

By comparing the previously reported transcriptome analysis with the DEGs in our study, we observed that few genes were uniformly regulated by IFNT between EECs and *in vivo* uteri. In the co-culture system of bovine EECs and stromal cells, the authors identified 222 upregulated DEGs in bovine EECs by comparing IFNT-treated and untreated groups; of which 96 of 222 appeared in our DEG list and all showed upregulation (22). We also compared our data with the DEGs in the receptive uterus vs. unreceptive uterus reported by Zhang et al. (23), because there are few reports on the transcriptome of IFNT-treated cells. There are 5 of 73 upregulated and 53 of 737 downregulated DEGs in the goat receptive uterus vs. unreceptive uterus were matched to our DEG list, and only 2 of 5 and 42 of 53 of upregulated and downregulated DEGs, respectively, were consistent with the gene regulation trends in our data (23). Similarly, in an earlier report, Gray et al. used microarray analysis to identify 45 genes specifically regulated by IFNT in the ovine uterus, but it only yielded 10 of 45 genes, consistent with our data (24).

Using bioinformatics analysis, we found that the significant enrichment and higher number of GO terms induced by IFNT were involved mainly in the regulation of enzyme activity, protein modification, and defense responses. Notably, ISG15 is involved in the formation of nuclease activity and ubiquitin-like modification, which once executes its functional interaction with the DNA helicase and substrate protein and regulates the immune response by altering the genome stability and protein function (25, 26). However, *in vivo* studies have shown that energy metabolism is the most important enriched function in the ruminant receptive uterus (23, 27), and were also predicted to potentially improve pregnancy outcomes (28). KEGG analysis in this study also demonstrated that defense response pathways, such as RIG-I (DDX58) like receptor, NF-κB, and JAK/STAT pathway, were remarkably enriched pathway, which is similar to the results of GO analysis.

It has been previously reported that the JAK/STAT signaling pathway mediates the formation of endometrial receptivity through the induction of IFNT (29). Therefore, we selected the JAK/STAT signaling pathway among the DEGs enriched for validation. In line with these expectations, IFNT markedly induced the transcription of STATs and phosphorylation of STAT1. However, the expression of p-STAT3 was negatively regulated by IFNT from 6 to 24 h, which is consistent with the maintenance time of the phosphorylation state of STAT3 (30, 31). Previous *in vivo* studies on STAT expression were limited to transcriptional and unphosphorylated protein levels (17, 18). Therefore, we focused on the localization and expression of p-STATs in the goat endometrium. As described in the literature, the expression of classical ISGs in LE/sGE is inhibited by interferon regulatory factor 2 (IRF2) (31). Similarly, STAT1 and p-STAT1 were hardly expressed in LE/sGE cells on P15. However, in the GE, STAT1 and p-STAT1 were completely undetectable, which is contrary to previous studies reporting that classical ISGs are strongly expressed in the ovine endometrial GE during the peri-implantation period (32, 33). The mechanism underlying this abnormality requires further study. Moreover, on P15, we observed that STAT3 was widely expressed in LE/GE/sGE cells, but we did not detect the expression of p-STAT3. This result may be related to the formation of unphosphorylated STAT3. Unphosphorylated STAT3 is also induced by IFN and can, in turn, induce the transcription of some classical ISGs through nuclear translocation (34).

In summary, these findings provide the first evidence of transcriptome changes in STATs via IFNT stimulation *in vitro*. We demonstrated that the distribution of STAT1 and STAT3 changes temporally and spatially in the goat endometrium during embryo implantation and that STAT1 and STAT3 may exert different effects on goat embryo implantation.

## Data Availability Statement

The datasets presented in this study can be found in online repositories. The names of the repository/repositories and accession number(s) can be found in the article/Supplementary Material.

## Ethics Statement

The experimental and surgical procedures were conducted according to protocols approved by the Committee for the Ethics on Animal Care and Experiments at Northwest A&F University.

## Author Contributions

HL and PL designed the study. HL, CW, and ZL performed the experiments. HL and ZL analyzed the data. HL wrote the manuscript and revised by PL, AW, and YJ. All authors contributed to the article and approved the submitted version.

## Funding

This study was supported by Key R&D Program project of the Ningxia Hui Autonomous Region (2021BBF02037) and the National Natural Science Foundation of China (Grant No. 32172934).

## Conflict of Interest

The authors declare that the research was conducted in the absence of any commercial or financial relationships that could be construed as a potential conflict of interest.

## Publisher's Note

All claims expressed in this article are solely those of the authors and do not necessarily represent those of their affiliated organizations, or those of the publisher, the editors and the reviewers. Any product that may be evaluated in this article, or claim that may be made by its manufacturer, is not guaranteed or endorsed by the publisher.

## References

[1] Bazer FW Seo H Wu G Johnson GA. Interferon tau: Influences on growth and development of the conceptus. Theriogenology. (2020) 150:75–83. 10.1016/j.theriogenology.2020.01.069 32088030

[2] Biase FH Hue I Dickinson SE Jaffrezic F Laloe D Lewin HA. Fine-tuned adaptation of embryo-endometrium pairs at implantation revealed by transcriptome analyses in Bos taurus. PLoS Biol. (2019) 17:e3000046. 10.1371/journal.pbio.3000046 30978203 PMC6481875

[3] Reese ST Franco GA Poole RK Hood R Fernadez Montero L Oliveira Filho RV. Pregnancy loss in beef cattle: a meta-analysis. Anim Reprod Sci. (2020) 212:106251. 10.1016/j.anireprosci.2019.106251 31864492

[4] Wiltbank MC Baez GM Garcia-Guerra A Toledo MZ Monteiro PL Melo LF. Pivotal periods for pregnancy loss during the first trimester of gestation in lactating dairy cows. Theriogenology. (2016) 86:239–53. 10.1016/j.theriogenology.2016.04.037 27238438

[5] Salilew-Wondim D Hölker M Rings F Ghanem N Ulas-Cinar M Peippo J. Bovine pretransfer endometrium and embryo transcriptome fingerprints as predictors of pregnancy success after embryo transfer. Physiol Genomics. (2010) 42:201–18. 10.1152/physiolgenomics.00047.2010 20388838

[6] Clemente M Lopez-Vidriero I O'Gaora P Mehta JP Forde N Gutierrez-Adan A. Transcriptome changes at the initiation of elongation in the bovine conceptus. Biol Reprod. (2011) 85:285–95. 10.1095/biolreprod.111.091587 21508349

[7] Boruszewska D Kowalczyk-Zieba I Sinderewicz E Grycmacher K Staszkiewicz J Woclawek-Potocka I. The effect of lysophosphatidic acid together with interferon tau on the global transcriptomic profile in bovine endometrial cells. Theriogenology. (2017) 92:111–20. 10.1016/j.theriogenology.2017.01.021 28237325

[8] Rabaglino MB Kadarmideen HN. Machine learning approach to integrated endometrial transcriptomic datasets reveals biomarkers predicting uterine receptivity in cattle at seven days after estrous. Sci Rep. (2020) 10:16981. 10.1038/s41598-020-72988-3 33046742 PMC7550564

[9] Roberts RM. Interferon-tau, a Type 1 interferon involved in maternal recognition of pregnancy. Cytokine Growth Factor Rev. (2007) 18:403–8. 10.1016/j.cytogfr.2007.06.010 17662642 PMC2000448

[10] Dorniak P Bazer FW Spencer TE. Physiology and endocrinology symposium: biological role of interferon tau in endometrial function and conceptus elongation. J Anim Sci. (2013) 91:1627–38. 10.2527/jas.2012-5845 23097402

[11] Leung S Qureshi SA Kerr IM Darnell JE Jr Stark GR. Role of STAT2 in the alpha interferon signaling pathway. Mol Cell Biol. (1995) 15:1312–7. 10.1128/MCB.15.3.1312 7532278 PMC230354

[12] Quelle FW Thierfelder W Witthuhn BA Tang B Cohen S Ihle JN. Phosphorylation, and activation of the DNA binding activity of purified Stat1 by the Janus protein-tyrosine kinases and the epidermal growth factor receptor. J Biol Chem. (1995) 270:20775–80. 10.1074/jbc.270.35.20775 7657660

[13] Kessler DS Levy DE Darnell JE Jr. Two interferon-induced nuclear factors bind a single promoter element in interferon-stimulated genes. Proc Natl Acad Sci USA. (1988) 85:8521–5. 10.1073/pnas.85.22.8521 2460869 PMC282490

[14] Beadling C Guschin D Witthuhn BA Ziemiecki A Ihle JN Kerr IM. Activation of JAK kinases and STAT proteins by interleukin-2 and interferon alpha, but not the T cell antigen receptor, in human T lymphocytes. Embo J. (1994) 13:5605–15. 10.1002/j.1460-2075.1994.tb06898.x 7988557 PMC395525

[15] Shao H Xu X Mastrangelo MA Jing N Cook RG Legge GB. Structural requirements for signal transducer and activator of transcription 3 binding to phosphotyrosine ligands containing the YXXQ motif. J Biol Chem. (2004) 279:18967–73. 10.1074/jbc.M314037200 14966128

[16] Stancato LF David M Carter-Su C Larner AC Pratt WB. Preassociation of STAT1 with STAT2 and STAT3 in separate signalling complexes prior to cytokine stimulation. J Biol Chem. (1996) 271:4134–7. 10.1074/jbc.271.8.4134 8626752

[17] Choi Y Johnson GA Burghardt RC Berghman LR Joyce MM Taylor KM. Interferon regulatory factor-two restricts expression of interferon-stimulated genes to the endometrial stroma and glandular epithelium of the ovine uterus. Biol Reprod. (2001) 65:1038–49. 10.1095/biolreprod65.4.1038 11566724

[18] Vitorino C Eozenou C Healey GD Forde N Reinaud P Chebrout M. Analysis of STAT1 expression and biological activity reveals interferon-tau-dependent STAT1-regulated SOCS genes in the bovine endometrium. Reprod Fertil Dev. (2016) 28:459–74. 10.1071/RD14034 25116692

[19] Hiraoka T Hirota Y Fukui Y Gebril M Kaku T Aikawa S. Differential roles of uterine epithelial and stromal STAT3 coordinate uterine receptivity and embryo attachment. Sci Rep. (2020) 10:15523. 10.1038/s41598-020-72640-0 32968170 PMC7511330

[20] Teng CB Diao HL Ma H Cong J Yu H Ma XH. Signal transducer and activator of transcription 3 (Stat3) expression and activation in rat uterus during early pregnancy. Reproduction. (2004) 128:197–205. 10.1530/rep.1.00053 15280559

[21] Zhang YY Wang AH Wu QX Sheng HX Jin YP. Establishment and characteristics of immortal goat endometrial epithelial cells and stromal cells with hTERT. J Anim Vet Adv. (2010) 9:2738–47. 10.3923/javaa.2010.2738.2747

[22] Chaney HL Grose LF Charpigny G Behura SK Sheldon IM Cronin JG. Conceptus-induced, interferon tau-dependent gene expression in bovine endometrial epithelial and stromal cells^†^. Biol Reprod. (2021) 104:669–83. 10.1093/biolre/ioaa226 33330929

[23] Zhang L Liu X Liu J Ma L Zhou Z Song Y. The developmental transcriptome landscape of receptive endometrium during embryo implantation in dairy goats. Gene. (2017) 633:82–95. 10.1016/j.gene.2017.08.026 28866083

[24] Gray CA Abbey CA Beremand PD Choi Y Farmer JL Adelson DL. Identification of endometrial genes regulated by early pregnancy, progesterone, and interferon tau in the ovine uterus. Biol Reprod. (2006) 74:383–94. 10.95/biolreprod.105.046656 16251498

[25] Raso MC Djoric N Walser F Hess S Schmid FM Burger S. Interferon-stimulated gene 15 accelerates replication fork progression inducing chromosomal breakage. J Cell Biol. (2020) 219:e202002175. 10.1083/jcb.202002175 32597933 PMC7401800

[26] Zhu C Li J Tian C Qin M Wang Z Shi B. Proteomic analysis of ISGylation in immortalized porcine alveolar macrophage cell lines induced by type I interferon. Vaccines. (2021) 9:164. 10.3390/vaccines9020164 33671165 PMC7922875

[27] Binelli M Scolari SC Pugliesi G Van Hoeck V Gonella-Diaza AM Andrade SC. The transcriptome signature of the receptive bovine uterus determined at early gestation. PLoS ONE. (2015) 10:e0122874. 10.1371/journal.pone.0122874 25849079 PMC4388694

[28] He A Zou Y Wan C Zhao J Zhang Q Yao Z. The role of transcriptomic biomarkers of endometrial receptivity in personalized embryo transfer for patients with repeated implantation failure. J Transl Med. (2021) 19:176. 10.1186/s12967-021-02837-y 33910562 PMC8082865

[29] Binelli M Subramaniam P Diaz T Johnson GA Hansen TR Badinga L. Bovine interferon-tau stimulates the Janus kinase-signal transducer and activator of transcription pathway in bovine endometrial epithelial cells. Biol Reprod. (2001) 64:654–65. 10.1095/biolreprod 11159370

[30] Stewart MD Johnson GA Vyhlidal CA Burghardt RC Safe SH Yu-Lee LY. Interferon-tau activates multiple signal transducer and activator of transcription proteins and has complex effects on interferon-responsive gene transcription in ovine endometrial epithelial cells. Endocrinology. (2001) 142:98–107. 10.1210/endo.142.1.7891 11145571

[31] Bazer FW Burghardt RC Johnson GA Spencer TE Wu G. Mechanisms for the establishment and maintenance of pregnancy: synergies from scientific collaborations. Biol Reprod. (2018) 99:225–41. 10.1093/biolre/ioy047 29462279 PMC6044348

[32] Song G Fleming JA Kim J Spencer TE Bazer FW. Pregnancy, and interferon tau regulate DDX58 and PLSCR1 in the ovine uterus during the peri-implantation period. Reproduction. (2011) 141:127–38. 10.1530/REP-10-0348 20926691

[33] Joyce MM White FJ Burghardt RC Muñiz JJ Spencer TE Bazer FW. Interferon stimulated gene 15 conjugates to endometrial cytosolic proteins and is expressed at the uterine-placental interface throughout pregnancy in sheep. Endocrinology. (2005) 146:675–84. 10.1210/en.2004-1224 15528302

[34] Cheon H Stark GR. Unphosphorylated STAT1 prolongs the expression of interferon-induced immune regulatory genes. Proc Natl Acad Sci USA. (2009) 106:9373–8. 10.1073/pnas.0903487106 19478064 PMC2688000

