# Supplementary material for: Transcriptomic Analysis of STAT1/3 in the Goat Endometrium During Embryo Implantation
Source: Front Vet Sci. 2021 Oct 14;8:757759. doi: 10.3389/fvets.2021.757759 (PMC8551392; doi:10.3389/fvets.2021.757759)
Supplement: Supplementary file 1 [file Table_1.docx]

**Table. S1 Primer Sequences**

| Gene ID | Gene name | Sequences (5’-3’) | The size of amplimers/bp |
| --- | --- | --- | --- |
| 102189170 | STAT1 | GAAGAGTCCACCAACGGGAG  ATCACCACGACGGGTAGAGA | 197 |
| 102190301 | STAT2 | TCAGAACTGGCAGGAAGCTG  GGGCCTGAATGTCCCGATAG | 167 |
| 102186154 | STAT3 | ACCATTGACCTGCCGATGTC  CTCCATGTCGAACGTGAGGG | 120 |
| 100860872 | GAPDH | TGCCCGTTCGACAGATAGC  ACGATGTCCACTTTGCCAGTA | 145 |

**Table. S2 Differentially Expressed Genes**

| transcript_id | gene_name | IFN_tau_FPKM | Control_FPKM | log2.foldchange. | pvalue | qvalue |
| --- | --- | --- | --- | --- | --- | --- |
| NM_001285571.1 | FKBP8 | 3.9872 | 0.6299 | 2.662200015 | 5.54E-05 | 0.050128 |
| NM_001285589.1 | EIF4EBP1 | 25.1781 | 36.0527 | -0.517938884 | 0.010032 | 0.301266 |
| NM_001285619.1 | SCD | 7.2736 | 4.6734 | 0.638187207 | 0.040585 | 0.426138 |
| NM_001285656.1 | RHEB | 88.1574 | 98.4094 | -0.158714939 | 0.041011 | 0.426138 |
| NM_001285670.1 | KITLG | 11.9529 | 7.7850 | 0.618584032 | 0.019753 | 0.33981 |
| NM_001285712.1 | GPX4 | 101.4520 | 122.6740 | -0.274032292 | 0.000927 | 0.154796 |
| NM_001285751.1 | NR1H3 | 7.6497 | 10.3173 | -0.431583129 | 0.025248 | 0.372995 |
| NM_001286965.1 | RAB1A | 100.8569 | 110.7642 | -0.135183206 | 0.002153 | 0.180463 |
| NM_001286991.1 | SLC39A13 | 11.6741 | 12.6646 | -0.117490567 | 0.010583 | 0.301764 |
| NM_001287560.1 | RAB18 | 56.4960 | 68.0008 | -0.267401804 | 0.015204 | 0.321836 |
| NM_001290120.1 | OAZ2 | 26.7141 | 33.0162 | -0.305570542 | 0.003356 | 0.196891 |
| NM_001291299.1 | PEG10 | 9.8226 | 10.4550 | -0.090022032 | 0.024118 | 0.365232 |
| NM_001314352.1 | CTSF | 25.5986 | 31.1389 | -0.282653904 | 0.006496 | 0.25356 |
| XM_005674717.3 | USP16 | 0.7619 | 0.9509 | -0.319721025 | 0.048013 | 0.445351 |
| XM_005674718.3 | USP16 | 1.0238 | 1.4370 | -0.489174834 | 0.04628 | 0.440825 |
| XM_005674830.2 | COL8A1 | 324.5417 | 373.8423 | -0.204025913 | 0.001892 | 0.180463 |
| XM_005674883.3 | DCBLD2 | 181.1579 | 168.5292 | 0.104249367 | 0.04004 | 0.422228 |
| XM_005674936.3 | ATP6V1A | 23.1275 | 26.8400 | -0.214778731 | 0.042366 | 0.430122 |
| XM_005675028.3 | PARP9 | 21.6000 | 2.4245 | 3.155255652 | 0.006285 | 0.25356 |
| XM_005675030.3 | PARP14 | 112.0684 | 13.2179 | 3.083811991 | 0.001912 | 0.180463 |
| XM_005675044.2 | UMPS | 11.9529 | 9.8337 | 0.281553928 | 0.016375 | 0.331741 |
| XM_005675123.3 | CLDN1 | 4.7405 | 0.5922 | 3.000935326 | 0.025447 | 0.374212 |
| XM_005675140.3 | RTP4 | 44.8774 | 5.9743 | 2.909142149 | 0.002106 | 0.180463 |
| XM_005675308.3 | TNFSF10 | 28.6929 | 0.1889 | 7.246939587 | 0.008155 | 0.276602 |
| XM_005675335.3 | PHC3 | 0.6270 | 3.1847 | -2.344501095 | 0.01558 | 0.324885 |
| XM_005675483.3 | XRN1 | 29.8541 | 24.1528 | 0.305737674 | 0.04705 | 0.443552 |
| XM_005675545.3 | ANAPC13 | 2.7417 | 4.8787 | -0.831410339 | 0.021063 | 0.346176 |
| XM_005675654.3 | LOC102186806 | 25.9322 | 29.3828 | -0.180228873 | 0.012053 | 0.310278 |
| XM_005675862.3 | OSGEPL1 | 3.6526 | 4.9611 | -0.441720987 | 0.020874 | 0.345787 |
| XM_005675863.3 | ASNSD1 | 0.3681 | 11.8915 | -5.013526315 | 0.001051 | 0.166924 |
| XM_005675864.3 | ASNSD1 | 8.4580 | 1.9160 | 2.142188878 | 0.001738 | 0.180463 |
| XM_005675869.3 | COL3A1 | 4127.2835 | 5049.6795 | -0.290999281 | 0.046304 | 0.440825 |
| XM_005676158.2 | RBM43 | 17.4499 | 4.7876 | 1.865849286 | 0.017301 | 0.334473 |
| XM_005676390.3 | LOC102176567 | 67.0758 | 49.2269 | 0.446345916 | 0.013934 | 0.315345 |
| XM_005676607.3 | FARSB | 36.1316 | 35.2677 | 0.034915992 | 0.018429 | 0.336847 |
| XM_005676659.3 | SP140 | 29.8453 | 12.5241 | 1.252802667 | 0.008578 | 0.284651 |
| XM_005676684.3 | AK2 | 20.8471 | 18.7746 | 0.151064579 | 0.010962 | 0.302822 |
| XM_005676857.3 | FUCA1 | 41.0627 | 46.5959 | -0.182374527 | 0.016771 | 0.332248 |
| XM_005677089.2 | FAM78B | 12.1523 | 17.3307 | -0.512108873 | 0.010531 | 0.301764 |
| XM_005677223.2 | COPA | 95.2628 | 107.8595 | -0.17916875 | 0.027434 | 0.381178 |
| XM_005677238.3 | TAGLN2 | 151.3985 | 155.6889 | -0.040314793 | 0.011245 | 0.304169 |
| XM_005677348.3 | GLMP | 11.6237 | 14.4284 | -0.311844235 | 0.040026 | 0.422228 |
| XM_005677369.3 | KIAA0907 | 4.2165 | 2.5891 | 0.703577321 | 0.022225 | 0.351287 |
| XM_005677414.2 | FLAD1 | 1.8791 | 1.8988 | -0.015066785 | 0.01534 | 0.323733 |
| XM_005677502.3 | S100A4 | 890.5897 | 1166.6272 | -0.389510663 | 0.017864 | 0.335634 |
| XM_005677603.3 | SELENBP1 | 185.8741 | 239.3036 | -0.364516679 | 0.019759 | 0.33981 |
| XM_005677634.3 | MLLT11 | 8.7781 | 11.5163 | -0.391696197 | 0.034307 | 0.402471 |
| XM_005677661.3 | GOLPH3L | 9.2870 | 11.2769 | -0.280078227 | 0.017862 | 0.335634 |
| XM_005677745.3 | POLR3C | 0.6061 | 0.6954 | -0.19819432 | 0.049979 | 0.448354 |
| XM_005677800.3 | GDAP2 | 5.5404 | 3.9827 | 0.476231928 | 0.012876 | 0.312555 |
| XM_005677808.3 | TTF2 | 12.9642 | 5.8460 | 1.149023718 | 0.016235 | 0.331741 |
| XM_005677838.3 | CSDE1 | 29.4298 | 45.7226 | -0.635629952 | 0.032895 | 0.3972 |
| XM_005677840.3 | CSDE1 | 128.4427 | 146.8595 | -0.193311946 | 0.002283 | 0.180463 |
| XM_005677969.3 | GPSM2 | 0.5071 | 2.0683 | -2.028021438 | 0.04509 | 0.437516 |
| XM_005678025.3 | COL11A1 | 10.4696 | 13.3826 | -0.354158644 | 0.027459 | 0.381178 |
| XM_005678157.3 | BCL10 | 16.1339 | 14.3388 | 0.17016909 | 0.033789 | 0.401488 |
| XM_005678196.3 | IFI44 | 32.7079 | 1.1009 | 4.892904063 | 0.002101 | 0.180463 |
| XM_005678197.3 | IFI44 | 13.9065 | 0.4835 | 4.846235253 | 0.006681 | 0.256734 |
| XM_005678249.3 | IFI44L | 94.2556 | 3.8867 | 4.599966966 | 0.00274 | 0.189392 |
| XM_005678304.3 | PDE4B | 7.2334 | 0.0302 | 7.901842382 | 0.00018 | 0.090478 |
| XM_005678500.3 | EFCAB14 | 16.2656 | 14.2100 | 0.194915871 | 0.005619 | 0.242877 |
| XM_005678522.2 | GPBP1L1 | 10.1709 | 12.6957 | -0.319883326 | 0.000568 | 0.138845 |
| XM_005678523.3 | GPBP1L1 | 16.0973 | 13.0751 | 0.299992528 | 0.023379 | 0.36378 |
| XM_005678573.3 | ELOVL1 | 11.8645 | 14.5729 | -0.296636404 | 0.02084 | 0.345787 |
| XM_005678674.3 | UTP11 | 29.4723 | 34.0280 | -0.207363396 | 0.012255 | 0.310879 |
| XM_005678918.3 | SAMD9 | 63.4657 | 3.2062 | 4.307035964 | 0.000429 | 0.133828 |
| XM_005678919.3 | SAMD9 | 25.9613 | 0.9387 | 4.789491363 | 2.56E-05 | 0.040045 |
| XM_005679049.3 | GPNMB | 31.5705 | 34.6384 | -0.133794287 | 0.006419 | 0.25356 |
| XM_005679058.3 | LOC102176015 | 13.5799 | 9.1994 | 0.561866573 | 0.031799 | 0.393623 |
| XM_005679153.3 | WNT2 | 20.5418 | 24.5027 | -0.254380253 | 0.016183 | 0.331741 |
| XM_005679283.3 | HNRNPA2B1 | 28.4914 | 24.4970 | 0.217918407 | 0.030185 | 0.387394 |
| XM_005679284.3 | HNRNPA2B1 | 97.0357 | 82.5959 | 0.232445628 | 0.030559 | 0.389228 |
| XM_005679305.3 | STEAP2 | 33.1813 | 39.9408 | -0.267492833 | 0.042783 | 0.430871 |
| XM_005679337.3 | BLVRA | 8.2052 | 10.3606 | -0.336495187 | 0.000827 | 0.149701 |
| XM_005679398.3 | AASS | 5.6996 | 1.2591 | 2.178515446 | 0.038917 | 0.41858 |
| XM_005679507.3 | MTPN | 409.9637 | 357.1140 | 0.199111593 | 0.010949 | 0.302822 |
| XM_005679508.3 | CHRM2 | 13.4348 | 8.6799 | 0.630218663 | 0.005393 | 0.242877 |
| XM_005679542.3 | CLEC5A | 5.2451 | 2.2395 | 1.227811086 | 0.01748 | 0.335382 |
| XM_005679654.2 | CDK5 | 13.8010 | 20.3827 | -0.562566141 | 0.005632 | 0.242877 |
| XM_005679813.3 | DCN | 286.6599 | 346.9547 | -0.275407135 | 0.005671 | 0.243402 |
| XM_005680008.3 | ARF3 | 21.1532 | 17.6070 | 0.264731054 | 0.03885 | 0.41858 |
| XM_005680287.3 | INHBE | 12.0191 | 15.3790 | -0.355640301 | 0.018304 | 0.336847 |
| XM_005680367.3 | MYL6B | 7.3674 | 8.8581 | -0.265846933 | 0.047049 | 0.443552 |
| XM_005680381.3 | DGKA | 4.7470 | 1.9991 | 1.247676499 | 0.031904 | 0.393623 |
| XM_005680479.3 | SLC25A3 | 179.4062 | 208.8975 | -0.219565748 | 0.024013 | 0.364979 |
| XM_005680657.3 | TXN2 | 12.2712 | 17.0211 | -0.472040338 | 0.028379 | 0.381178 |
| XM_005680701.3 | IPO8 | 12.5841 | 11.7441 | 0.099661515 | 0.041115 | 0.426138 |
| XM_005680737.3 | ITPR2 | 6.8332 | 4.9766 | 0.457411793 | 0.042229 | 0.429917 |
| XM_005680810.3 | HEBP1 | 21.5072 | 29.3607 | -0.449065227 | 0.014033 | 0.315345 |
| XM_005681117.3 | XRCC6 | 20.2619 | 18.2007 | 0.154774912 | 0.028924 | 0.383912 |
| XM_005681119.3 | SNU13 | 18.4009 | 21.0490 | -0.193975905 | 0.028934 | 0.383912 |
| XM_005681134.3 | POLDIP3 | 35.2713 | 38.4165 | -0.123230007 | 0.027302 | 0.381178 |
| XM_005681455.3 | MED28 | 22.3909 | 24.8909 | -0.152704308 | 0.045265 | 0.437516 |
| XM_005681489.3 | TBC1D19 | 8.6114 | 9.8764 | -0.19774789 | 0.020132 | 0.341604 |
| XM_005681520.3 | LIAS | 6.3528 | 7.9674 | -0.326722768 | 0.002894 | 0.193219 |
| XM_005681551.3 | UCHL1 | 162.2532 | 186.2245 | -0.198795645 | 0.008045 | 0.276356 |
| XM_005681792.3 | SCARB2 | 89.8822 | 79.9106 | 0.169647609 | 0.004977 | 0.234012 |
| XM_005681813.3 | ANTXR2 | 109.6145 | 95.6893 | 0.196008505 | 0.010948 | 0.302822 |
| XM_005681827.3 | ENOPH1 | 13.0900 | 14.3681 | -0.134410472 | 0.00899 | 0.289542 |
| XM_005681847.3 | PLAC8 | 11.5267 | 0.2328 | 5.629903824 | 0.027847 | 0.381178 |
| XM_005681881.3 | NUDT9 | 23.3521 | 25.6038 | -0.132804914 | 0.019776 | 0.33981 |
| XM_005682134.3 | USE1 | 18.5132 | 21.3837 | -0.207951056 | 0.020997 | 0.345787 |
| XM_005682285.3 | JUNB | 58.9712 | 71.2242 | -0.272356615 | 0.015086 | 0.321836 |
| XM_005682339.3 | UBL5 | 27.2874 | 33.9581 | -0.315518779 | 0.029874 | 0.38622 |
| XM_005682392.3 | ELOF1 | 13.2349 | 17.5428 | -0.40653536 | 0.026079 | 0.377377 |
| XM_005682534.3 | CD70 | 3.9271 | 6.1513 | -0.647430834 | 0.004589 | 0.22224 |
| XM_005682692.3 | ALDH7A1 | 26.1784 | 30.3315 | -0.212442782 | 0.04822 | 0.446267 |
| XM_005682906.3 | ATP5D | 26.7734 | 31.4016 | -0.230039439 | 0.030712 | 0.389638 |
| XM_005682946.3 | DDX46 | 15.8258 | 12.6411 | 0.32415216 | 0.012842 | 0.312555 |
| XM_005682969.3 | FAM13B | 1.2339 | 3.4658 | -1.489928634 | 0.043707 | 0.433856 |
| XM_005683003.3 | SIL1 | 3.8572 | 6.2624 | -0.699143394 | 0.045082 | 0.437516 |
| XM_005683019.3 | DNAJC18 | 9.0960 | 11.7635 | -0.371010036 | 0.010281 | 0.301266 |
| XM_005683040.3 | SRA1 | 39.9570 | 48.7722 | -0.287608234 | 0.006502 | 0.25356 |
| XM_005683183.3 | GPX3 | 19.4332 | 21.7853 | -0.164832857 | 0.010062 | 0.301266 |
| XM_005683188.3 | GM2A | 40.7356 | 27.3681 | 0.573792941 | 0.032789 | 0.3972 |
| XM_005683370.3 | NUDCD2 | 16.6447 | 20.1591 | -0.276367232 | 0.036083 | 0.410838 |
| XM_005683400.3 | EDIL3 | 18.8765 | 22.5426 | -0.256058891 | 0.012541 | 0.312555 |
| XM_005683436.2 | SLF1 | 7.0424 | 4.8659 | 0.533359973 | 0.03588 | 0.40975 |
| XM_005683451.3 | ERAP1 | 20.9125 | 12.2908 | 0.766782697 | 0.001874 | 0.180463 |
| XM_005683453.3 | ERAP2 | 35.6494 | 26.2117 | 0.44367013 | 0.021746 | 0.350408 |
| XM_005683520.3 | HAND2 | 17.9563 | 20.9868 | -0.22499632 | 0.008592 | 0.284651 |
| XM_005683566.3 | DDX58 | 250.6186 | 3.7534 | 6.061166906 | 8.36E-05 | 0.066252 |
| XM_005683677.3 | TMEM261 | 9.6716 | 12.7103 | -0.394174128 | 0.0284 | 0.381178 |
| XM_005683693.3 | CD274 | 10.1057 | 2.2444 | 2.170782987 | 0.042527 | 0.430833 |
| XM_005683802.3 | PSAT1 | 51.9154 | 61.0240 | -0.233214174 | 0.011219 | 0.304169 |
| XM_005683829.3 | STOML2 | 32.1252 | 35.7138 | -0.152775046 | 0.031883 | 0.393623 |
| XM_005683864.3 | GLIPR2 | 69.8067 | 78.9705 | -0.177948516 | 0.004109 | 0.210245 |
| XM_005684281.3 | FAM206A | 8.4929 | 11.7807 | -0.472089321 | 0.025939 | 0.376446 |
| XM_005684357.3 | PSMD5 | 15.7020 | 17.2734 | -0.137606684 | 0.002282 | 0.180463 |
| XM_005684442.3 | HTR1B | 84.8778 | 61.0620 | 0.475111703 | 0.008612 | 0.284651 |
| XM_005684486.3 | NCOA7 | 2.5860 | 1.7348 | 0.575962174 | 0.047206 | 0.443552 |
| XM_005684572.3 | CDK19 | 16.9713 | 15.5988 | 0.121661518 | 0.028837 | 0.383912 |
| XM_005684718.3 | TBX18 | 23.1324 | 19.5674 | 0.241459901 | 0.025753 | 0.374829 |
| XM_005684807.3 | IFNGR1 | 12.2613 | 10.8372 | 0.178123443 | 0.032385 | 0.395454 |
| XM_005684899.2 | PPIL4 | 10.2935 | 12.0929 | -0.232418555 | 0.010666 | 0.302074 |
| XM_005685131.3 | RPL4 | 686.4191 | 736.7904 | -0.102164597 | 0.009356 | 0.293554 |
| XM_005685224.3 | IRF9 | 16.8812 | 1.3559 | 3.638075326 | 0.034953 | 0.405298 |
| XM_005685227.3 | RNF31 | 5.3028 | 3.1633 | 0.745331392 | 0.033234 | 0.398184 |
| XM_005685228.3 | PSME2 | 3.8957 | 1.9091 | 1.029026556 | 0.01657 | 0.331741 |
| XM_005685278.3 | ABHD4 | 6.3413 | 7.3586 | -0.214655756 | 0.011832 | 0.309246 |
| XM_005685359.3 | TOX4 | 15.7864 | 16.8246 | -0.091886465 | 0.012985 | 0.312555 |
| XM_005685363.3 | SUPT16H | 40.9253 | 38.5675 | 0.085606437 | 0.032031 | 0.394529 |
| XM_005685406.3 | APEX1 | 20.8284 | 12.7457 | 0.708536695 | 0.004432 | 0.218418 |
| XM_005685407.2 | APEX1 | 0.4445 | 9.1816 | -4.368587735 | 0.015569 | 0.324885 |
| XM_005685488.3 | C10H15orf57 | 3.2548 | 5.5530 | -0.770683562 | 0.019663 | 0.33981 |
| XM_005685523.3 | EHD4 | 52.6067 | 36.2490 | 0.537306505 | 0.001473 | 0.173757 |
| XM_005685652.3 | GNG2 | 41.4974 | 48.2915 | -0.218748798 | 0.003785 | 0.204863 |
| XM_005686014.3 | SLC39A9 | 12.0339 | 13.6928 | -0.186315598 | 0.046658 | 0.441549 |
| XM_005686045.2 | PSEN1 | 21.3039 | 19.1442 | 0.154211057 | 0.039282 | 0.420992 |
| XM_005686092.3 | ACYP1 | 5.4872 | 6.6319 | -0.273351579 | 0.048307 | 0.446267 |
| XM_005686317.3 | LOXL3 | 29.8407 | 37.5890 | -0.333031794 | 0.001626 | 0.179548 |
| XM_005686332.3 | WBP1 | 3.7833 | 7.7220 | -1.029324509 | 0.046491 | 0.440859 |
| XM_005686372.3 | PRADC1 | 8.7117 | 9.5719 | -0.135849761 | 0.026426 | 0.378059 |
| XM_005686393.2 | DPY30 | 4.6955 | 9.4788 | -1.013430019 | 0.032276 | 0.395454 |
| XM_005686623.2 | PPP4R3B | 27.1047 | 32.4984 | -0.261824779 | 0.000143 | 0.083979 |
| XM_005686626.3 | PNPT1 | 35.2777 | 8.3253 | 2.083187733 | 0.002506 | 0.188329 |
| XM_005686651.2 | GCC2 | 13.4906 | 12.4699 | 0.11350999 | 0.036663 | 0.411631 |
| XM_005686885.3 | NRBP1 | 48.0359 | 52.7092 | -0.133941929 | 0.009984 | 0.301266 |
| XM_005686978.3 | WDR35 | 5.5675 | 7.2261 | -0.376198827 | 0.043327 | 0.43296 |
| XM_005687185.3 | TRUB2 | 6.6160 | 7.9744 | -0.269422559 | 0.033167 | 0.398184 |
| XM_005687412.3 | MTRF1 | 17.2941 | 24.1666 | -0.482740032 | 0.00223 | 0.180463 |
| XM_005687598.3 | EBPL | 6.7353 | 8.2348 | -0.28999337 | 0.028564 | 0.382086 |
| XM_005687668.3 | LMO7 | 46.1527 | 58.9804 | -0.353818432 | 0.005514 | 0.242877 |
| XM_005687853.3 | UPF2 | 13.4671 | 10.6748 | 0.33523016 | 0.022195 | 0.351287 |
| XM_005687928.3 | COMMD3 | 14.2496 | 19.4618 | -0.44973126 | 0.049572 | 0.448354 |
| XM_005687952.3 | SEPHS1 | 5.1040 | 3.0428 | 0.746213549 | 0.011594 | 0.309168 |
| XM_005687973.3 | FAM188A | 5.5874 | 0.7014 | 2.993830756 | 0.013626 | 0.315345 |
| XM_005687974.2 | FAM188A | 3.8786 | 19.3149 | -2.316102733 | 0.005117 | 0.235447 |
| XM_005688175.2 | TRMT6 | 17.0523 | 14.4931 | 0.23460246 | 0.012963 | 0.312555 |
| XM_005688313.3 | ZBP1 | 10.7355 | 0.2716 | 5.304540069 | 0.012656 | 0.312555 |
| XM_005688330.3 | PSMF1 | 20.8355 | 6.3293 | 1.718926746 | 0.000252 | 0.108821 |
| XM_005688331.3 | PSMF1 | 7.2640 | 2.0506 | 1.824703711 | 0.010674 | 0.302074 |
| XM_005688572.2 | FITM2 | 2.2336 | 4.5128 | -1.014646967 | 0.041203 | 0.426444 |
| XM_005688851.3 | SQLE | 29.7594 | 25.1851 | 0.240775686 | 0.016399 | 0.331741 |
| XM_005688873.3 | HAS2 | 110.7993 | 58.4200 | 0.923415446 | 0.010582 | 0.301764 |
| XM_005688982.3 | TMEM68 | 2.7190 | 3.5053 | -0.366449797 | 0.002773 | 0.18989 |
| XM_005689064.3 | RDH10 | 24.5753 | 15.6653 | 0.649630288 | 0.040801 | 0.426138 |
| XM_005689136.3 | EXT1 | 77.3934 | 68.9285 | 0.167110723 | 0.035868 | 0.40975 |
| XM_005689154.3 | NUDCD1 | 12.2627 | 9.9476 | 0.301861478 | 0.004719 | 0.226117 |
| XM_005689227.3 | TP53INP1 | 29.5559 | 41.8143 | -0.500547944 | 0.003658 | 0.204863 |
| XM_005689436.3 | CRYAB | 9.8620 | 12.8657 | -0.383573162 | 0.005631 | 0.242877 |
| XM_005689491.3 | TAGLN | 576.7824 | 709.2017 | -0.298168848 | 0.027741 | 0.381178 |
| XM_005689565.3 | HSPA8 | 94.6078 | 70.6142 | 0.421999663 | 0.041989 | 0.42988 |
| XM_005689681.3 | MRVI1 | 5.6492 | 10.2989 | -0.866375513 | 0.021411 | 0.348286 |
| XM_005689702.3 | SWAP70 | 33.2225 | 38.6463 | -0.218168799 | 0.013623 | 0.315345 |
| XM_005689800.3 | LOC102170165 | 11.4504 | 1.1737 | 3.286237524 | 0.045312 | 0.437516 |
| XM_005689802.3 | LOC102170446 | 9.9198 | 2.0777 | 2.25532192 | 0.017029 | 0.332366 |
| XM_005689839.2 | TRIM68 | 6.3186 | 4.3702 | 0.531909021 | 0.01073 | 0.302723 |
| XM_005689840.3 | TRIM68 | 1.0693 | 3.1055 | -1.538130123 | 0.021982 | 0.351287 |
| XM_005689846.3 | TRIM21 | 17.8964 | 5.5218 | 1.69646555 | 0.002292 | 0.180463 |
| XM_005689973.2 | POLD3 | 10.5212 | 6.6517 | 0.661514083 | 0.013468 | 0.315345 |
| XM_005690299.3 | FAM111B | 14.0388 | 2.7970 | 2.327470094 | 0.002049 | 0.180463 |
| XM_005690382.3 | ZC3H11A | 0.9474 | 3.1595 | -1.737588815 | 0.000764 | 0.147423 |
| XM_005690630.2 | KIFAP3 | 28.9169 | 33.7379 | -0.222460797 | 0.026371 | 0.377878 |
| XM_005690654.2 | PIGC | 4.0409 | 4.7507 | -0.233467508 | 0.006716 | 0.256734 |
| XM_005690657.2 | TNFSF18 | 7.0766 | 2.7253 | 1.376633104 | 0.009016 | 0.289542 |
| XM_005690719.3 | LZIC | 0.0004 | 2.9540 | -12.86608284 | 0.001339 | 0.173757 |
| XM_005690784.3 | AURKAIP1 | 28.4458 | 28.7798 | -0.016840314 | 0.031558 | 0.393455 |
| XM_005690795.3 | ISG15 | 522.4805 | 2.4142 | 7.757693673 | 0.001601 | 0.179046 |
| XM_005690953.2 | DHX9 | 16.4586 | 14.4946 | 0.183326794 | 0.020975 | 0.345787 |
| XM_005690990.2 | PLA2G4A | 118.1703 | 103.8208 | 0.186771143 | 0.023878 | 0.364979 |
| XM_005691043.3 | HSD11B1 | 11.7585 | 14.1323 | -0.265291662 | 0.035835 | 0.40975 |
| XM_005691166.3 | TRIM2 | 2.4435 | 2.7613 | -0.176390761 | 0.014327 | 0.31684 |
| XM_005691200.3 | EDNRA | 30.6268 | 23.8840 | 0.35875212 | 0.02347 | 0.363951 |
| XM_005691420.3 | P2RX4 | 20.9159 | 17.3840 | 0.266842193 | 0.002713 | 0.189392 |
| XM_005691488.3 | LOC102190983 | 443.5706 | 3.5979 | 6.945852443 | 0.000148 | 0.083979 |
| XM_005691787.3 | SF3B3 | 51.3932 | 45.8590 | 0.164373395 | 0.045317 | 0.437516 |
| XM_005691789.3 | AARS | 44.9215 | 50.2301 | -0.161145743 | 0.028234 | 0.381178 |
| XM_005691820.3 | DYNLRB2 | 3.0178 | 4.6675 | -0.629135228 | 0.020504 | 0.345612 |
| XM_005691822.3 | CENPN | 7.1211 | 4.9588 | 0.522094393 | 0.047991 | 0.445351 |
| XM_005692178.3 | PRMT7 | 8.4863 | 10.2815 | -0.276832106 | 0.001706 | 0.180463 |
| XM_005692196.3 | PSMD7 | 45.5251 | 39.3163 | 0.211533674 | 0.029835 | 0.38622 |
| XM_005692231.3 | CMTR2 | 22.3725 | 3.9814 | 2.490361354 | 0.003886 | 0.204863 |
| XM_005692245.2 | FAAP24 | 5.4488 | 3.6644 | 0.572371076 | 0.003016 | 0.193715 |
| XM_005692524.3 | KCNK6 | 16.3698 | 14.5805 | 0.166992612 | 0.016102 | 0.331741 |
| XM_005692690.3 | PLEKHA4 | 9.6896 | 2.2287 | 2.12025043 | 0.037018 | 0.411631 |
| XM_005692692.3 | PPP1R15A | 8.8476 | 11.8075 | -0.416349828 | 0.014673 | 0.31789 |
| XM_005692819.3 | EMP3 | 83.3601 | 44.0397 | 0.920551214 | 0.036527 | 0.410919 |
| XM_005693123.3 | CLTC | 116.5348 | 144.5991 | -0.311296769 | 0.043363 | 0.43296 |
| XM_005693232.3 | SUZ12 | 16.8393 | 14.4111 | 0.224657783 | 0.024446 | 0.368053 |
| XM_005693242.2 | LOC102184087 | 2.1113 | 2.4874 | -0.2365402 | 0.042251 | 0.429917 |
| XM_005693243.2 | LOC102184087 | 87.3815 | 112.1435 | -0.35994647 | 0.031869 | 0.393623 |
| XM_005693322.3 | TSR1 | 16.8860 | 11.6809 | 0.531676253 | 0.019227 | 0.339544 |
| XM_005693352.3 | POLDIP2 | 13.5213 | 15.5290 | -0.199734553 | 0.029896 | 0.38622 |
| XM_005693418.3 | ANKFY1 | 16.3739 | 11.4611 | 0.514649616 | 0.00678 | 0.257299 |
| XM_005693425.3 | XAF1 | 12.7627 | 1.0624 | 3.586571729 | 0.000508 | 0.137526 |
| XM_005693467.3 | MED11 | 5.7935 | 7.9818 | -0.462269801 | 0.000219 | 0.103836 |
| XM_005693537.2 | TRAPPC1 | 6.3540 | 8.5846 | -0.434092666 | 0.00438 | 0.218418 |
| XM_005693653.3 | LUC7L3 | 11.3489 | 10.0009 | 0.182415667 | 0.032064 | 0.394529 |
| XM_005693666.3 | ACSF2 | 16.1987 | 20.2278 | -0.320463331 | 0.006421 | 0.25356 |
| XM_005693840.2 | NKIRAS2 | 7.9904 | 9.8800 | -0.306247341 | 0.014361 | 0.31684 |
| XM_005693881.3 | PTGES3L | 3.7381 | 5.6299 | -0.590804057 | 0.013044 | 0.312555 |
| XM_005693886.3 | IFI35 | 73.7786 | 22.2576 | 1.728904305 | 0.017581 | 0.335634 |
| XM_005693992.3 | DDX42 | 12.1271 | 9.5632 | 0.342674598 | 0.012925 | 0.312555 |
| XM_005694051.2 | PYCR1 | 19.6024 | 25.6955 | -0.39048687 | 0.002243 | 0.180463 |
| XM_005694380.3 | RFNG | 15.6268 | 16.9343 | -0.115928684 | 0.003277 | 0.196891 |
| XM_005694630.3 | TRAPPC13 | 8.2868 | 10.2050 | -0.300377302 | 0.024541 | 0.36856 |
| XM_005694641.3 | SREK1IP1 | 6.9601 | 8.7049 | -0.322726444 | 0.039811 | 0.422228 |
| XM_005694692.3 | IL6ST | 231.1957 | 188.3141 | 0.295973458 | 0.000577 | 0.138845 |
| XM_005694721.3 | ITGA2 | 8.3193 | 5.1595 | 0.689230746 | 0.026332 | 0.377878 |
| XM_005694730.3 | FGF10 | 17.3268 | 10.0516 | 0.785587325 | 0.037754 | 0.413911 |
| XM_005694925.3 | LYSMD4 | 0.3699 | 1.1004 | -1.572804496 | 0.007422 | 0.268073 |
| XM_005694942.3 | ISG20 | 50.1053 | 1.4949 | 5.066871297 | 0.012885 | 0.312555 |
| XM_005694967.3 | ABHD2 | 22.8484 | 18.8756 | 0.2755694 | 0.012352 | 0.312469 |
| XM_005694976.3 | LOC102191560 | 7.7728 | 10.3453 | -0.412466151 | 0.013101 | 0.312555 |
| XM_005695068.3 | ARNT2 | 1.0778 | 3.4737 | -1.688329548 | 0.009575 | 0.295948 |
| XM_005695137.3 | LOC102170823 | 7.4393 | 3.9005 | 0.931524553 | 0.047935 | 0.445351 |
| XM_005695149.3 | PML | 9.9468 | 4.6499 | 1.097014563 | 0.017396 | 0.334473 |
| XM_005695156.3 | PML | 4.3745 | 1.7358 | 1.333478952 | 0.019695 | 0.33981 |
| XM_005695408.3 | WARS | 41.4005 | 11.1633 | 1.890879304 | 0.021563 | 0.349258 |
| XM_005695506.3 | AZI2 | 4.8934 | 16.5106 | -1.754488141 | 0.034242 | 0.402471 |
| XM_005695707.2 | SUMF1 | 24.7059 | 27.6279 | -0.161270777 | 0.007531 | 0.268073 |
| XM_005695783.2 | PDHB | 30.1645 | 37.4899 | -0.31364914 | 0.024738 | 0.369683 |
| XM_005695819.3 | PDE12 | 9.6452 | 5.6476 | 0.772177071 | 0.028906 | 0.383912 |
| XM_005695886.3 | ALAS1 | 39.7662 | 61.6686 | -0.632991492 | 0.011762 | 0.309246 |
| XM_005695943.2 | UBA7 | 31.2275 | 4.1221 | 2.921368241 | 0.023455 | 0.363951 |
| XM_005695962.3 | GPX1 | 126.2871 | 145.5307 | -0.204615523 | 0.012782 | 0.312555 |
| XM_005695973.2 | IMPDH2 | 29.1486 | 33.1960 | -0.187582802 | 0.018971 | 0.339529 |
| XM_005696047.3 | LZTFL1 | 15.8452 | 19.7161 | -0.315325519 | 0.039391 | 0.421162 |
| XM_005696089.3 | SPCS1 | 20.3121 | 23.4563 | -0.207636249 | 0.006299 | 0.25356 |
| XM_005696413.3 | MCM3 | 23.2577 | 17.5452 | 0.406635553 | 0.022021 | 0.351287 |
| XM_005696445.3 | TAP1 | 29.0167 | 4.4876 | 2.692871228 | 0.008556 | 0.284651 |
| XM_005696446.3 | PSMB9 | 21.6766 | 2.2492 | 3.268632278 | 0.026197 | 0.377377 |
| XM_005696560.3 | NELFE | 20.9076 | 23.4694 | -0.166751347 | 0.006329 | 0.25356 |
| XM_005696612.3 | LOC102188814 | 168.0732 | 31.5853 | 2.41176507 | 0.002214 | 0.180463 |
| XM_005696631.3 | IER3 | 571.8469 | 744.9626 | -0.381539066 | 0.01786 | 0.335634 |
| XM_005697046.3 | RIOK3 | 42.1103 | 58.1827 | -0.466416901 | 0.027703 | 0.381178 |
| XM_005697060.3 | ROCK1 | 49.0931 | 48.5500 | 0.016046675 | 0.010123 | 0.301266 |
| XM_005697318.2 | LOC102181552 | 411.2495 | 282.2154 | 0.543217388 | 0.006455 | 0.25356 |
| XM_005697330.3 | SNRNP25 | 10.3906 | 13.5331 | -0.381222132 | 0.031756 | 0.393623 |
| XM_005697399.3 | MSRB1 | 19.7836 | 24.8005 | -0.326058525 | 0.005159 | 0.235447 |
| XM_005697580.3 | LYRM1 | 0.5465 | 1.3301 | -1.28310835 | 0.028379 | 0.381178 |
| XM_005697619.2 | LCMT1 | 15.5222 | 17.5340 | -0.175818913 | 0.037252 | 0.411631 |
| XM_005697749.3 | LOC102183030 | 6.7951 | 11.2929 | -0.73284941 | 0.048928 | 0.44826 |
| XM_005697752.3 | PSPH | 32.1608 | 38.8524 | -0.272698332 | 0.033359 | 0.39908 |
| XM_005697835.3 | CNPY4 | 19.3063 | 22.3647 | -0.212149947 | 0.040171 | 0.422518 |
| XM_005698076.3 | MOSPD3 | 6.1709 | 7.8309 | -0.343683863 | 0.012098 | 0.310278 |
| XM_005698192.3 | ACTA2 | 141.9442 | 250.5988 | -0.820054954 | 0.000318 | 0.125116 |
| XM_005698193.3 | CH25H | 8.9398 | 21.2919 | -1.251994362 | 0.000577 | 0.138845 |
| XM_005698194.3 | LOC102168687 | 841.9897 | 39.6285 | 4.409191977 | 0.000665 | 0.146847 |
| XM_005698197.3 | IFIT2 | 46.3430 | 2.7082 | 4.096940264 | 0.010368 | 0.301266 |
| XM_005698236.2 | HELLS | 19.2434 | 12.7155 | 0.597776325 | 0.005135 | 0.235447 |
| XM_005698317.3 | SLC25A28 | 16.0738 | 4.9646 | 1.694945314 | 0.016803 | 0.332248 |
| XM_005698331.2 | SLF2 | 16.2140 | 11.7185 | 0.468450498 | 0.029501 | 0.3854 |
| XM_005698432.3 | SLK | 17.7325 | 9.5671 | 0.890232123 | 0.003361 | 0.196891 |
| XM_005698468.3 | BBIP1 | 2.0530 | 4.3468 | -1.082190725 | 0.016932 | 0.332366 |
| XM_005698780.3 | DLC1 | 1.7239 | 0.4849 | 1.830045677 | 0.047233 | 0.443552 |
| XM_005698819.3 | MAK16 | 12.1745 | 8.9727 | 0.440250752 | 0.00324 | 0.196891 |
| XM_005698890.3 | PLAT | 32.9572 | 39.5770 | -0.264068909 | 0.04152 | 0.428254 |
| XM_005698926.3 | RARB | 5.8710 | 8.1425 | -0.471862416 | 0.033946 | 0.401488 |
| XM_005698959.3 | SPHAR | 5.9797 | 7.2625 | -0.280396724 | 0.016445 | 0.331741 |
| XM_005698968.3 | ARV1 | 10.5683 | 12.4584 | -0.237383567 | 0.025786 | 0.374829 |
| XM_005699006.3 | GNPAT | 27.0406 | 30.7821 | -0.186966321 | 0.006819 | 0.257299 |
| XM_005699130.3 | VPS26A | 46.0365 | 42.9746 | 0.099294024 | 0.036403 | 0.410838 |
| XM_005699196.3 | DNAJC9 | 15.3171 | 11.1331 | 0.460288927 | 0.019754 | 0.33981 |
| XM_005699200.3 | MRPS16 | 22.5083 | 23.6096 | -0.068916273 | 0.0422 | 0.429917 |
| XM_005699218.3 | CHCHD1 | 18.5003 | 20.9867 | -0.18192735 | 0.044727 | 0.437516 |
| XM_005699229.3 | ADK | 18.7499 | 22.4612 | -0.260551343 | 0.039764 | 0.422228 |
| XM_005699356.3 | PARG | 9.5541 | 7.7576 | 0.300513071 | 0.047358 | 0.443552 |
| XM_005699555.2 | VWA5A | 78.9847 | 69.1091 | 0.192696446 | 0.046088 | 0.440798 |
| XM_005699592.3 | STT3A | 157.8957 | 173.2560 | -0.133932641 | 0.04542 | 0.437574 |
| XM_005699764.3 | FEN1 | 12.3001 | 9.7466 | 0.335697262 | 0.037138 | 0.411631 |
| XM_005699776.3 | EEF1G | 343.9867 | 408.6354 | -0.248461479 | 0.008056 | 0.276356 |
| XM_005699837.3 | ATL3 | 112.2212 | 70.0061 | 0.680792137 | 0.002078 | 0.180463 |
| XM_005699896.3 | SSSCA1 | 6.0548 | 3.9298 | 0.623622582 | 0.004028 | 0.208709 |
| XM_005699918.3 | YIF1A | 39.1315 | 45.0836 | -0.204271276 | 0.007934 | 0.276055 |
| XM_005699920.3 | B4GAT1 | 6.9947 | 10.1281 | -0.534033811 | 0.00185 | 0.180463 |
| XM_005699935.3 | RBM4 | 5.2010 | 6.7606 | -0.378368128 | 0.003848 | 0.204863 |
| XM_005699945.3 | POLD4 | 10.7910 | 15.0616 | -0.481045303 | 0.006866 | 0.258008 |
| XM_005699947.3 | AIP | 7.7308 | 10.9386 | -0.500734258 | 0.011326 | 0.305265 |
| XM_005700083.3 | LOC102180655 | 195.8875 | 40.7400 | 2.265507946 | 0.001726 | 0.180463 |
| XM_005700085.3 | PSMD13 | 38.2613 | 40.5676 | -0.084444143 | 0.005456 | 0.242877 |
| XM_005700222.2 | ATG4A | 8.6805 | 11.5140 | -0.407538322 | 0.003791 | 0.204863 |
| XM_005700344.3 | CUL4B | 24.9183 | 12.4919 | 0.996218403 | 0.010212 | 0.301266 |
| XM_005700349.3 | C1GALT1C1 | 22.2416 | 17.7075 | 0.328895511 | 0.033839 | 0.401488 |
| XM_005700707.3 | IGBP1 | 15.9175 | 19.6844 | -0.306444953 | 0.030201 | 0.387394 |
| XM_005700713.3 | PJA1 | 8.9709 | 10.6301 | -0.244836404 | 0.00899 | 0.289542 |
| XM_005700756.3 | WDR45 | 10.1660 | 13.6348 | -0.423542269 | 0.013849 | 0.315345 |
| XM_005700868.3 | SMC1A | 16.3553 | 14.2957 | 0.194178982 | 0.003405 | 0.196891 |
| XM_005701415.3 | LOC102173932 | 166.3295 | 11.2604 | 3.884708733 | 0.002688 | 0.189392 |
| XM_005701416.3 | LOC102173932 | 36.5247 | 1.8880 | 4.273920445 | 0.02624 | 0.377377 |
| XM_005701596.3 | ARSA | 10.3373 | 11.7067 | -0.179476837 | 0.037607 | 0.413911 |
| XM_005702074.3 | LOC102185270 | 7.9788 | 12.0182 | -0.590983051 | 0.03072 | 0.389638 |
| XM_005709381.3 | SEPW1 | 30.5869 | 38.0346 | -0.314398473 | 0.001288 | 0.173757 |
| XM_005709406.3 | RPL6 | 375.0814 | 408.1126 | -0.12176338 | 0.009011 | 0.289542 |
| XM_005709424.3 | BLZF1 | 20.9521 | 22.9808 | -0.133335285 | 0.042089 | 0.42988 |
| XM_005709457.3 | TMEM69 | 6.6019 | 9.1333 | -0.468260482 | 0.036946 | 0.411631 |
| XM_005709548.3 | TRIM25 | 19.4315 | 7.2575 | 1.420855199 | 0.000655 | 0.146847 |
| XM_005709697.3 | HN1 | 14.7159 | 15.8074 | -0.103220049 | 0.015606 | 0.324885 |
| XM_013963262.2 | PARP9 | 23.3730 | 3.1946 | 2.871122199 | 0.01521 | 0.321836 |
| XM_013963273.2 | PARP9 | 8.8688 | 1.5918 | 2.478097005 | 0.031718 | 0.393623 |
| XM_013963581.2 | ATP6V0E2 | 8.6976 | 10.1819 | -0.227323983 | 0.036847 | 0.411631 |
| XM_013963668.2 | COPZ1 | 40.9936 | 48.0482 | -0.229086403 | 0.032383 | 0.395454 |
| XM_013963948.2 | RBMS2 | 10.7186 | 4.8838 | 1.134028874 | 0.030098 | 0.387339 |
| XM_013964616.2 | SLC30A9 | 26.3385 | 29.6191 | -0.16935711 | 0.044561 | 0.436832 |
| XM_013965021.2 | SMIM7 | 4.2321 | 5.3060 | -0.32625432 | 0.016956 | 0.332366 |
| XM_013965033.2 | TRIM59 | 1.4836 | 2.7063 | -0.867198148 | 0.040075 | 0.422228 |
| XM_013965444.2 | SPARC | 716.9600 | 903.4940 | -0.333622383 | 0.019825 | 0.33981 |
| XM_013965507.2 | CDC34 | 16.1958 | 17.6122 | -0.120961847 | 0.023931 | 0.364979 |
| XM_013965663.2 | TCERG1 | 1.3913 | 1.0949 | 0.345665764 | 0.010557 | 0.301764 |
| XM_013965775.2 | RASA1 | 23.6602 | 21.4700 | 0.140136753 | 0.035624 | 0.409403 |
| XM_013965842.2 | LOC102171788 | 2.5479 | 4.3750 | -0.780003945 | 0.031586 | 0.393455 |
| XM_013965949.2 | PDCD1LG2 | 2.8339 | 0.3749 | 2.918015612 | 0.040842 | 0.426138 |
| XM_013966322.2 | ACP1 | 19.7445 | 19.7160 | 0.002082585 | 0.031329 | 0.393455 |
| XM_013966327.2 | MB21D1 | 16.3306 | 4.3834 | 1.897459279 | 0.005592 | 0.242877 |
| XM_013966728.2 | PAPD4 | 5.7701 | 3.0599 | 0.915103625 | 0.038031 | 0.41402 |
| XM_013966733.2 | PAPD4 | 6.1044 | 3.8310 | 0.672132297 | 0.042029 | 0.42988 |
| XM_013967148.2 | ATP8B4 | 36.9158 | 17.0538 | 1.11414247 | 0.031178 | 0.392692 |
| XM_013967480.2 | HTRA2 | 6.6216 | 7.9035 | -0.255309704 | 0.016331 | 0.331741 |
| XM_013967602.2 | PREPL | 4.6680 | 8.5394 | -0.871309055 | 0.047504 | 0.443964 |
| XM_013967643.2 | IL36B | 4.8577 | 2.5749 | 0.915783262 | 0.003685 | 0.204863 |
| XM_013967752.2 | LOC106502633 | 3.6107 | 1.3740 | 1.393917952 | 0.016321 | 0.331741 |
| XM_013968297.2 | MYCBP2 | 18.9335 | 15.7863 | 0.262273684 | 0.026982 | 0.381178 |
| XM_013968320.2 | GPR180 | 20.7556 | 18.1547 | 0.193155941 | 0.04438 | 0.436384 |
| XM_013968519.2 | ITGA4 | 59.1490 | 55.5929 | 0.089452813 | 0.039674 | 0.422228 |
| XM_013968785.2 | SRXN1 | 32.7502 | 33.4386 | -0.030007282 | 0.020608 | 0.345612 |
| XM_013968798.2 | ZBP1 | 3.5055 | 0.0081 | 8.754810818 | 0.011638 | 0.309168 |
| XM_013969192.2 | VCPIP1 | 16.5721 | 9.8739 | 0.747067849 | 0.048392 | 0.446267 |
| XM_013969349.2 | EBAG9 | 6.1997 | 1.1152 | 2.474911932 | 0.023209 | 0.362383 |
| XM_013969634.2 | ZBED5 | 6.4021 | 5.6770 | 0.173418623 | 0.032591 | 0.39617 |
| XM_013969972.2 | FAM111B | 5.4228 | 0.5415 | 3.323953921 | 0.00315 | 0.196891 |
| XM_013970338.2 | LOC106501722 | 5.4989 | 3.9971 | 0.460201012 | 0.026253 | 0.377377 |
| XM_013970374.2 | QSOX1 | 17.3025 | 20.1423 | -0.219249114 | 0.039033 | 0.418823 |
| XM_013970957.2 | SDR42E1 | 0.9290 | 1.2729 | -0.454388376 | 0.01579 | 0.327964 |
| XM_013971139.2 | BMPR2 | 27.1520 | 27.0329 | 0.006338403 | 0.041761 | 0.429755 |
| XM_013971420.2 | ZNF428 | 6.2857 | 8.0543 | -0.357688555 | 0.019481 | 0.339544 |
| XM_013972340.2 | RNF213 | 61.2577 | 3.2130 | 4.252913291 | 0.000229 | 0.103836 |
| XM_013972341.2 | RNF213 | 5.8016 | 0.9744 | 2.573851531 | 0.002966 | 0.193219 |
| XM_013972342.2 | RNF213 | 4.2831 | 0.9313 | 2.201336644 | 0.011252 | 0.304169 |
| XM_013972568.2 | TMEM107 | 3.2609 | 6.5433 | -1.00473171 | 0.002119 | 0.180463 |
| XM_013972697.2 | NPM1 | 27.6440 | 21.8417 | 0.339884906 | 0.02389 | 0.364979 |
| XM_013972724.2 | ERBIN | 13.7005 | 13.7871 | -0.009094183 | 0.038037 | 0.41402 |
| XM_013972785.2 | OXCT1 | 28.3372 | 30.4097 | -0.101832405 | 0.040982 | 0.426138 |
| XM_013973120.2 | G2E3 | 12.4392 | 10.1823 | 0.288822433 | 0.039472 | 0.421533 |
| XM_013973146.2 | WDR76 | 10.2538 | 8.4839 | 0.273359962 | 0.047032 | 0.443552 |
| XM_013973217.2 | SP140 | 17.4426 | 6.2857 | 1.472461341 | 0.036429 | 0.410838 |
| XM_013973259.2 | LOC102186814 | 54.4535 | 10.4617 | 2.379903961 | 0.004286 | 0.216833 |
| XM_013973395.2 | AZI2 | 3.9776 | 0.0713 | 5.800996019 | 0.008027 | 0.276356 |
| XM_013973462.2 | STAC | 2.4536 | 4.9331 | -1.007579691 | 0.035362 | 0.40691 |
| XM_013973591.2 | RBM6 | 5.0945 | 2.8642 | 0.830831199 | 0.023623 | 0.364979 |
| XM_013974950.2 | ITPRIPL2 | 29.8535 | 22.3611 | 0.416905133 | 0.000395 | 0.133828 |
| XM_013975200.2 | CD247 | 1.8765 | 3.1779 | -0.760079436 | 0.040609 | 0.426138 |
| XM_013975632.2 | C28H10orf54 | 1.7132 | 3.9981 | -1.222646919 | 0.004206 | 0.214008 |
| XM_013975693.2 | LOC102191494 | 20.5426 | 17.3624 | 0.242648186 | 0.029954 | 0.386409 |
| XM_013976637.2 | POLA1 | 9.3962 | 7.1691 | 0.390285633 | 0.017737 | 0.335634 |
| XM_013976927.2 | LOC102179935 | 28.2249 | 5.7231 | 2.302087553 | 0.023331 | 0.363656 |
| XM_013977098.2 | JTB | 14.6930 | 16.1828 | -0.139335317 | 0.014456 | 0.31684 |
| XM_018038231.1 | WNT5A | 64.1308 | 53.0968 | 0.272392265 | 0.007326 | 0.267514 |
| XM_018038329.1 | PSMD6 | 38.8640 | 43.0617 | -0.14797136 | 0.016123 | 0.331741 |
| XM_018038356.1 | ARF4 | 152.6549 | 163.9288 | -0.102795363 | 0.002335 | 0.182254 |
| XM_018038506.1 | LOC102189920 | 59.6318 | 44.3701 | 0.426494424 | 0.042722 | 0.430833 |
| XM_018038615.1 | ABHD16A | 3.5197 | 0.6708 | 2.391585818 | 0.005895 | 0.25064 |
| XM_018038879.1 | LOC102176782 | 16.3371 | 12.6534 | 0.368628424 | 0.025107 | 0.372995 |
| XM_018038903.1 | TAP2 | 46.5298 | 16.7168 | 1.476858518 | 0.034004 | 0.401488 |
| XM_018038941.1 | LOC102177764 | 34.1703 | 39.1754 | -0.197205445 | 0.019865 | 0.33981 |
| XM_018038944.1 | CMTR1 | 37.6654 | 18.8259 | 1.000524373 | 0.002284 | 0.180463 |
| XM_018038945.1 | TUBB2A | 22.8191 | 24.4692 | -0.100724396 | 0.011175 | 0.304169 |
| XM_018038969.1 | MTCH1 | 24.6996 | 31.6842 | -0.359273422 | 0.012163 | 0.310278 |
| XM_018038990.1 | SSR1 | 168.1516 | 190.2971 | -0.178490528 | 0.023048 | 0.360483 |
| XM_018038994.1 | C23H6orf62 | 15.2844 | 11.4869 | 0.412069347 | 0.045149 | 0.437516 |
| XM_018038995.1 | C23H6orf62 | 24.1408 | 21.7580 | 0.149927068 | 0.048636 | 0.447154 |
| XM_018038997.1 | RIPK1 | 17.4511 | 12.3710 | 0.49636003 | 0.003895 | 0.204863 |
| XM_018039022.1 | DEK | 15.4170 | 11.5602 | 0.415347578 | 0.019436 | 0.339544 |
| XM_018039045.1 | DDX39B | 52.0658 | 44.4711 | 0.227468478 | 0.01391 | 0.315345 |
| XM_018039118.1 | CDKN1A | 76.6554 | 89.8537 | -0.229190899 | 0.049688 | 0.448354 |
| XM_018039129.1 | LOC108633505 | 15.5993 | 11.0163 | 0.50184226 | 0.049747 | 0.448354 |
| XM_018039179.1 | FBXO9 | 24.5352 | 31.3729 | -0.354669531 | 0.018549 | 0.336847 |
| XM_018039302.1 | TMEM170B | 9.8689 | 7.8937 | 0.322181714 | 0.028991 | 0.383912 |
| XM_018039367.1 | ZCCHC2 | 6.4374 | 2.7051 | 1.250787371 | 0.001444 | 0.173757 |
| XM_018039467.1 | OSBPL1A | 3.0279 | 4.7025 | -0.635123965 | 0.026937 | 0.381178 |
| XM_018039520.1 | MIB1 | 19.3586 | 21.9246 | -0.17957652 | 0.026669 | 0.380342 |
| XM_018039556.1 | TUBB6 | 70.8968 | 66.9333 | 0.082997337 | 0.04077 | 0.426138 |
| XM_018039664.1 | SS18 | 14.0043 | 7.0311 | 0.994047814 | 0.021207 | 0.346658 |
| XM_018039665.1 | SS18 | 24.1173 | 26.7480 | -0.149361528 | 0.023654 | 0.364979 |
| XM_018039702.1 | LOC108633807 | 3.8273 | 6.3414 | -0.72846898 | 0.006364 | 0.25356 |
| XM_018039769.1 | DTYMK | 4.6710 | 5.6355 | -0.270809162 | 0.014649 | 0.31789 |
| XM_018039839.1 | MPG | 8.3626 | 10.6409 | -0.347595853 | 0.001497 | 0.173757 |
| XM_018039949.1 | TMEM219 | 3.3578 | 4.4302 | -0.399848081 | 0.028545 | 0.382086 |
| XM_018039970.1 | RHBDL1 | 5.4833 | 7.9166 | -0.529849788 | 0.0195 | 0.339544 |
| XM_018039973.1 | STUB1 | 33.1137 | 39.5345 | -0.255681889 | 0.02921 | 0.3854 |
| XM_018040081.1 | POLR3K | 14.8691 | 16.1026 | -0.114976868 | 0.003187 | 0.196891 |
| XM_018040166.1 | SLX1A | 3.8457 | 5.0889 | -0.404131012 | 0.016921 | 0.332366 |
| XM_018040185.1 | TUFM | 13.7323 | 19.5846 | -0.512147934 | 0.018859 | 0.338196 |
| XM_018040295.1 | ZNF771 | 6.3497 | 6.2394 | 0.025283619 | 0.040097 | 0.422228 |
| XM_018040333.1 | NME4 | 6.7557 | 6.9590 | -0.042764595 | 0.021318 | 0.347849 |
| XM_018040379.1 | PCOLCE | 119.9846 | 142.4028 | -0.247128263 | 0.031361 | 0.393455 |
| XM_018040594.1 | NTAN1 | 36.5603 | 38.8393 | -0.087239452 | 0.01931 | 0.339544 |
| XM_018040705.1 | NDUFB10 | 43.2162 | 50.1099 | -0.213522413 | 0.027077 | 0.381178 |
| XM_018040763.1 | LOC102175358 | 13.6571 | 38.1706 | -1.482808301 | 0.003329 | 0.196891 |
| XM_018040767.1 | CCZ1 | 13.3982 | 14.5555 | -0.119523994 | 0.015372 | 0.323733 |
| XM_018040776.1 | TSC22D4 | 5.0851 | 6.8315 | -0.42593986 | 0.00144 | 0.173757 |
| XM_018040798.1 | HCFC1R1 | 5.8181 | 9.8111 | -0.753859842 | 0.013115 | 0.312555 |
| XM_018040803.1 | C25H16orf52 | 10.0420 | 7.4903 | 0.422949036 | 0.043734 | 0.433856 |
| XM_018040824.1 | PLOD3 | 24.7885 | 29.8436 | -0.267749779 | 0.047932 | 0.445351 |
| XM_018040890.1 | NUBP2 | 4.8683 | 2.7318 | 0.833579328 | 0.035307 | 0.406796 |
| XM_018040932.1 | GDE1 | 1.1043 | 9.7145 | -3.137046366 | 0.020867 | 0.345787 |
| XM_018041067.1 | RMI2 | 8.8516 | 6.3001 | 0.490573633 | 0.016742 | 0.332248 |
| XM_018041185.1 | METTL10 | 1.5355 | 0.5522 | 1.475382645 | 0.041055 | 0.426138 |
| XM_018041259.1 | INPP5A | 9.5405 | 12.0019 | -0.331116306 | 0.018087 | 0.33628 |
| XM_018041260.1 | IFIT5 | 152.6750 | 40.3003 | 1.921600186 | 0.001207 | 0.17009 |
| XM_018041262.1 | IFIT3 | 198.4703 | 6.3216 | 4.972484747 | 0.004427 | 0.218418 |
| XM_018041272.1 | ZMYM6 | 2.2854 | 1.9005 | 0.266094464 | 0.043951 | 0.434516 |
| XM_018041274.1 | ECHS1 | 45.1151 | 50.4375 | -0.160885643 | 0.039904 | 0.422228 |
| XM_018041300.1 | MMS19 | 1.1885 | 2.2379 | -0.912951333 | 0.031453 | 0.393455 |
| XM_018041471.1 | MCMBP | 22.8079 | 20.2292 | 0.173094956 | 0.009125 | 0.290979 |
| XM_018041472.1 | MCMBP | 6.4645 | 5.9456 | 0.120715678 | 0.011219 | 0.304169 |
| XM_018041549.1 | BNIP3 | 13.9037 | 12.0850 | 0.202243238 | 0.027648 | 0.381178 |
| XM_018041573.1 | BUB3 | 22.3173 | 18.7944 | 0.247860426 | 0.000516 | 0.137526 |
| XM_018041598.1 | OBFC1 | 5.0408 | 2.3912 | 1.07591404 | 0.022048 | 0.351287 |
| XM_018041710.1 | RGS10 | 29.2493 | 30.6151 | -0.0658424 | 0.018303 | 0.336847 |
| XM_018041835.1 | GTF2E2 | 0.2050 | 2.4435 | -3.575476432 | 0.006288 | 0.25356 |
| XM_018041999.1 | NR1D2 | 32.6658 | 30.2639 | 0.110179639 | 0.049387 | 0.448354 |
| XM_018042025.1 | NRG1 | 9.9335 | 3.3901 | 1.550987089 | 0.01738 | 0.334473 |
| XM_018042027.1 | NRG1 | 12.5122 | 7.6618 | 0.707576968 | 0.010975 | 0.302822 |
| XM_018042112.1 | CDKN2AIP | 6.4037 | 1.8109 | 1.822214606 | 0.020303 | 0.343091 |
| XM_018042148.1 | SIRT1 | 9.3246 | 7.9888 | 0.223051239 | 0.04374 | 0.433856 |
| XM_018042170.1 | ANXA7 | 5.0867 | 5.5898 | -0.136046121 | 0.021203 | 0.346658 |
| XM_018042171.1 | ANXA7 | 6.6546 | 6.9199 | -0.056399274 | 0.042105 | 0.42988 |
| XM_018042212.1 | JMJD1C | 6.3293 | 3.5805 | 0.821860797 | 0.024051 | 0.364979 |
| XM_018042213.1 | JMJD1C | 8.8637 | 4.4284 | 1.001142782 | 0.019534 | 0.339544 |
| XM_018042341.1 | SAR1A | 125.8465 | 144.6331 | -0.200732042 | 0.030408 | 0.388754 |
| XM_018042345.1 | CCSER2 | 1.4761 | 1.2395 | 0.251997486 | 0.034057 | 0.401592 |
| XM_018042380.1 | CCDC6 | 21.3925 | 14.1684 | 0.5944336 | 0.002276 | 0.180463 |
| XM_018042435.1 | VDAC2 | 88.4329 | 96.7377 | -0.129494747 | 0.002223 | 0.180463 |
| XM_018042451.1 | LYST | 37.1793 | 28.6904 | 0.373934583 | 0.023935 | 0.364979 |
| XM_018042452.1 | LYST | 4.1618 | 7.1583 | -0.782411352 | 0.044285 | 0.435923 |
| XM_018042476.1 | FAM213A | 0.3730 | 1.3658 | -1.872453937 | 0.021174 | 0.346658 |
| XM_018042526.1 | TOMM20 | 59.5827 | 61.1648 | -0.037808731 | 0.04711 | 0.443552 |
| XM_018042612.1 | TMEM26 | 34.6752 | 24.8175 | 0.482546271 | 0.02712 | 0.381178 |
| XM_018042692.1 | EML3 | 7.9211 | 10.0294 | -0.340455282 | 0.002526 | 0.188329 |
| XM_018042697.1 | B3GAT3 | 13.9261 | 15.6944 | -0.172462921 | 0.002469 | 0.188329 |
| XM_018042698.1 | POLR2L | 72.1979 | 80.3049 | -0.153530906 | 0.038199 | 0.415287 |
| XM_018042913.1 | MACROD1 | 11.0068 | 12.9710 | -0.236904785 | 0.026226 | 0.377377 |
| XM_018042918.1 | ALDH3B1 | 7.0718 | 10.3397 | -0.548043717 | 0.045156 | 0.437516 |
| XM_018042977.1 | GSK3B | 11.5035 | 13.0303 | -0.179800638 | 0.042612 | 0.430833 |
| XM_018042996.1 | CEP295 | 3.3339 | 2.0914 | 0.67274813 | 0.015554 | 0.324885 |
| XM_018043034.1 | IRF7 | 14.3832 | 0.4862 | 4.886747052 | 0.001459 | 0.173757 |
| XM_018043042.1 | CDK2AP2 | 9.1578 | 11.5364 | -0.333119029 | 0.00767 | 0.270265 |
| XM_018043203.1 | SLC15A3 | 20.6273 | 5.5503 | 1.893915597 | 0.014227 | 0.31684 |
| XM_018043326.1 | ATL3 | 86.7126 | 34.7048 | 1.321104677 | 0.047181 | 0.443552 |
| XM_018043394.1 | PPP1CA | 37.8725 | 41.9152 | -0.14632351 | 0.005224 | 0.236551 |
| XM_018043423.1 | SLC36A4 | 28.3019 | 23.0179 | 0.298142015 | 0.017528 | 0.335593 |
| XM_018043445.1 | LOC108634238 | 90.9981 | 20.7770 | 2.130848368 | 0.009048 | 0.289542 |
| XM_018043446.1 | LOC106501751 | 17.3031 | 0.4231 | 5.35380166 | 0.005099 | 0.235447 |
| XM_018043455.1 | RAB1B | 26.8994 | 34.5544 | -0.361297746 | 0.032527 | 0.395921 |
| XM_018043458.1 | FAM89B | 0.9077 | 4.2728 | -2.234847702 | 0.001209 | 0.17009 |
| XM_018043534.1 | RHOD | 4.9123 | 7.1769 | -0.546955803 | 0.001831 | 0.180463 |
| XM_018043651.1 | UBA1 | 1.7118 | 3.4229 | -0.999670155 | 0.037063 | 0.411631 |
| XM_018043790.1 | TAF9B | 8.0891 | 5.8392 | 0.470223955 | 0.001839 | 0.180463 |
| XM_018043812.1 | ZFX | 0.0604 | 1.6932 | -4.809997214 | 0.033651 | 0.400979 |
| XM_018043901.1 | AP1S2 | 40.1623 | 49.6306 | -0.305390206 | 0.02087 | 0.345787 |
| XM_018043941.1 | CXHXorf38 | 15.6692 | 3.8696 | 2.017687347 | 0.006454 | 0.25356 |
| XM_018044165.1 | ORC1 | 5.7874 | 3.8425 | 0.590841946 | 0.011676 | 0.309168 |
| XM_018044177.1 | ACSL4 | 164.1360 | 124.4700 | 0.399093616 | 0.035869 | 0.40975 |
| XM_018044185.1 | FAM127A | 4.5771 | 6.1865 | -0.434692289 | 0.032328 | 0.395454 |
| XM_018044186.1 | LOC108634461 | 5.0703 | 6.9691 | -0.458902029 | 0.02777 | 0.381178 |
| XM_018044349.1 | UBL4A | 12.7526 | 16.9313 | -0.40890275 | 0.034745 | 0.40443 |
| XM_018044458.1 | LOC106503400 | 25.7135 | 31.1727 | -0.277756039 | 0.020307 | 0.343091 |
| XM_018044473.1 | LOC102191395 | 11.5766 | 13.5653 | -0.228704918 | 0.017854 | 0.335634 |
| XM_018044478.1 | LOC102185735 | 4.2801 | 2.6617 | 0.685309086 | 0.006918 | 0.258895 |
| XM_018044621.1 | DKC1 | 15.7217 | 12.2893 | 0.355356575 | 0.028879 | 0.383912 |
| XM_018044699.1 | LOC102173185 | 14.0278 | 0.9089 | 3.947955234 | 0.002818 | 0.190429 |
| XM_018044784.1 | LOC102184867 | 14.4024 | 5.1500 | 1.483666026 | 0.036045 | 0.410838 |
| XM_018044816.1 | MRPL40 | 15.4350 | 17.2862 | -0.163420127 | 0.04836 | 0.446267 |
| XM_018044862.1 | DGCR6L | 11.3836 | 13.4852 | -0.244420552 | 0.017733 | 0.335634 |
| XM_018044903.1 | MFAP2 | 44.2113 | 53.9679 | -0.287687904 | 0.027187 | 0.381178 |
| XM_018044958.1 | TMCO3 | 9.4846 | 11.5262 | -0.281255502 | 0.001987 | 0.180463 |
| XM_018044963.1 | LAMP1 | 72.0552 | 79.2843 | -0.137932693 | 0.019887 | 0.33981 |
| XM_018045063.1 | NAA10 | 23.7567 | 25.6047 | -0.108072435 | 0.015161 | 0.321836 |
| XM_018045143.1 | PLP2 | 79.8501 | 92.4659 | -0.211627672 | 0.009784 | 0.300358 |
| XM_018045263.1 | LOC102169471 | 25.2167 | 26.3920 | -0.065721134 | 0.036183 | 0.410838 |
| XM_018045316.1 | SHISA4 | 13.1987 | 14.8839 | -0.173349596 | 0.022055 | 0.351287 |
| XM_018045354.1 | RPL10 | 517.5743 | 541.4258 | -0.064997599 | 0.010294 | 0.301266 |
| XM_018045379.1 | LOC108635087 | 17.7223 | 26.8494 | -0.599327222 | 0.039904 | 0.422228 |
| XM_018045388.1 | LOC108635102 | 38.1525 | 28.0992 | 0.441251014 | 0.03266 | 0.396477 |
| XM_018045422.1 | LOC108635156 | 52.1673 | 56.4489 | -0.113799343 | 0.048046 | 0.445351 |
| XM_018045578.1 | KPNA1 | 17.7285 | 20.9115 | -0.23822762 | 0.007824 | 0.273581 |
| XM_018045665.1 | DTX3L | 57.3378 | 12.7805 | 2.165544906 | 0.007494 | 0.268073 |
| XM_018045708.1 | LOC102170144 | 67.0308 | 19.0865 | 1.812272085 | 0.02563 | 0.374367 |
| XM_018045709.1 | LOC102170144 | 17.6282 | 0.0316 | 9.123426793 | 0.029545 | 0.3854 |
| XM_018045710.1 | LOC102174264 | 36.5862 | 3.4016 | 3.426998235 | 0.010792 | 0.302822 |
| XM_018045771.1 | MYLK | 6.4811 | 9.8347 | -0.601643623 | 0.021001 | 0.345787 |
| XM_018045779.1 | BCAR3 | 18.9896 | 16.7146 | 0.184097498 | 0.014912 | 0.320767 |
| XM_018045781.1 | GCLM | 8.5995 | 31.5163 | -1.873781222 | 0.000165 | 0.08797 |
| XM_018045789.1 | ABCD3 | 11.7904 | 13.3860 | -0.183102175 | 0.023658 | 0.364979 |
| XM_018045791.1 | F3 | 90.4858 | 82.2837 | 0.137085025 | 0.044473 | 0.436821 |
| XM_018045853.1 | ITGB5 | 4.8790 | 6.5889 | -0.433454574 | 0.037799 | 0.413911 |
| XM_018045879.1 | LOC108633298 | 5.7993 | 8.7693 | -0.596581669 | 0.016594 | 0.331741 |
| XM_018045914.1 | DDX20 | 24.7180 | 20.8230 | 0.247381543 | 0.041127 | 0.426138 |
| XM_018045958.1 | CD58 | 9.5763 | 5.3312 | 0.845007516 | 0.035838 | 0.40975 |
| XM_018045972.1 | LOC102176008 | 13.7271 | 15.2721 | -0.153878208 | 0.017748 | 0.335634 |
| XM_018045984.1 | PHGDH | 27.6308 | 33.9822 | -0.298501706 | 0.003226 | 0.196891 |
| XM_018046005.1 | LOC102188370 | 6.5957 | 2.9943 | 1.139328247 | 0.021636 | 0.349258 |
| XM_018046028.1 | PLEKHO1 | 9.8091 | 13.6293 | -0.474519306 | 0.036344 | 0.410838 |
| XM_018046187.1 | C3H1orf43 | 4.4052 | 6.3061 | -0.517540629 | 0.003905 | 0.204863 |
| XM_018046197.1 | ADAR | 18.5258 | 3.3215 | 2.479631824 | 0.000359 | 0.133828 |
| XM_018046300.1 | CRABP2 | 13.5109 | 17.4454 | -0.368716486 | 0.04504 | 0.437516 |
| XM_018046307.1 | HDGF | 54.2765 | 60.1106 | -0.147291205 | 0.006719 | 0.256734 |
| XM_018046525.1 | ATP13A3 | 77.9287 | 41.8764 | 0.896018454 | 0.011832 | 0.309246 |
| XM_018046531.1 | ATP13A3 | 84.9168 | 47.9962 | 0.823129865 | 0.045657 | 0.438929 |
| XM_018046608.1 | CCDC50 | 50.5056 | 54.0524 | -0.097914769 | 0.019243 | 0.339544 |
| XM_018046696.1 | NUB1 | 4.1381 | 0.6710 | 2.624663627 | 0.011001 | 0.302822 |
| XM_018046842.1 | FAM131B | 12.5397 | 14.2267 | -0.182088686 | 0.020911 | 0.345787 |
| XM_018046976.1 | WASL | 12.7900 | 11.5874 | 0.142460126 | 0.027355 | 0.381178 |
| XM_018047025.1 | NUDCD3 | 11.4223 | 13.2022 | -0.208927817 | 0.001542 | 0.176793 |
| XM_018047071.1 | MPP6 | 2.4098 | 2.8782 | -0.256283916 | 0.011822 | 0.309246 |
| XM_018047105.1 | MTURN | 9.7403 | 13.6313 | -0.484875979 | 0.040747 | 0.426138 |
| XM_018047145.1 | 7-Sep | 2.3536 | 0.7892 | 1.576407961 | 0.039356 | 0.421162 |
| XM_018047186.1 | MET | 9.6827 | 5.2641 | 0.879222709 | 0.043155 | 0.43296 |
| XM_018047229.1 | PSMD2 | 70.4422 | 73.7836 | -0.066860559 | 0.001391 | 0.173757 |
| XM_018047232.1 | NAMPT | 22.0850 | 7.4004 | 1.577386645 | 0.034398 | 0.402471 |
| XM_018047234.1 | SYPL1 | 62.2059 | 48.9089 | 0.346953804 | 0.012062 | 0.310278 |
| XM_018047559.1 | RAB21 | 45.2433 | 41.0242 | 0.141231099 | 0.011178 | 0.304169 |
| XM_018047587.1 | CSRP2 | 14.2036 | 20.3943 | -0.52191043 | 0.006223 | 0.25356 |
| XM_018047597.1 | ZMAT3 | 29.9896 | 35.8620 | -0.257992054 | 0.041668 | 0.429294 |
| XM_018047616.1 | ATP2B1 | 12.8855 | 9.6063 | 0.42369757 | 0.007374 | 0.268073 |
| XM_018047618.1 | LUM | 51.3013 | 64.3173 | -0.326209964 | 0.01156 | 0.309168 |
| XM_018047625.1 | SOCS2 | 39.9857 | 31.5164 | 0.343381043 | 0.004969 | 0.234012 |
| XM_018047658.1 | CALCOCO1 | 4.9415 | 8.0326 | -0.700913049 | 0.033886 | 0.401488 |
| XM_018047660.1 | ATP5G2 | 79.4402 | 102.2242 | -0.363796464 | 0.043937 | 0.434516 |
| XM_018047680.1 | C5H12orf10 | 10.1658 | 12.1395 | -0.255991317 | 0.017737 | 0.335634 |
| XM_018047701.1 | FNDC3B | 34.4253 | 10.8067 | 1.67154383 | 0.022744 | 0.356348 |
| XM_018047709.1 | EIF4B | 59.7545 | 72.0790 | -0.270530692 | 0.017938 | 0.335928 |
| XM_018047710.1 | EIF4B | 93.7423 | 113.8939 | -0.280918348 | 0.01166 | 0.309168 |
| XM_018047730.1 | NR4A1 | 4.5713 | 6.8537 | -0.584289943 | 0.037879 | 0.41402 |
| XM_018047755.1 | SMAGP | 17.4359 | 11.6399 | 0.582979097 | 0.022094 | 0.351287 |
| XM_018047793.1 | DIP2B | 21.6886 | 17.6442 | 0.297740487 | 0.025181 | 0.372995 |
| XM_018047837.1 | SPATS2 | 16.9917 | 20.5780 | -0.276275313 | 0.032115 | 0.394618 |
| XM_018047881.1 | SLC48A1 | 6.9925 | 10.3251 | -0.562283071 | 0.002717 | 0.189392 |
| XM_018047899.1 | ANO6 | 17.8113 | 12.5182 | 0.5087651 | 0.018775 | 0.337348 |
| XM_018047988.1 | RASSF3 | 33.5531 | 30.7236 | 0.127099671 | 0.033901 | 0.401488 |
| XM_018047989.1 | TBK1 | 14.5841 | 11.4299 | 0.35158495 | 0.025601 | 0.374367 |
| XM_018047998.1 | SLC16A7 | 6.0772 | 7.7220 | -0.345571887 | 0.001181 | 0.17009 |
| XM_018048002.1 | TSFM | 33.2746 | 39.8231 | -0.259186273 | 0.02727 | 0.381178 |
| XM_018048046.1 | LOC108636012 | 25.6269 | 30.3616 | -0.244589253 | 0.029644 | 0.38551 |
| XM_018048069.1 | STAT2 | 36.5139 | 10.0196 | 1.86562871 | 0.005174 | 0.235447 |
| XM_018048109.1 | MMP19 | 40.4123 | 44.3326 | -0.133573615 | 0.043942 | 0.434516 |
| XM_018048122.1 | BLOC1S1 | 27.6549 | 33.9603 | -0.296312561 | 0.033475 | 0.399933 |
| XM_018048126.1 | PDCD10 | 12.0227 | 10.9186 | 0.138973744 | 0.03679 | 0.411631 |
| XM_018048130.1 | ELK3 | 88.5510 | 61.3097 | 0.530392686 | 0.002231 | 0.180463 |
| XM_018048135.1 | TMPO | 4.6653 | 1.9266 | 1.275925036 | 0.030514 | 0.389209 |
| XM_018048179.1 | PARPBP | 6.4650 | 15.8932 | -1.297680936 | 0.028041 | 0.381178 |
| XM_018048180.1 | NUP37 | 15.1664 | 19.9081 | -0.392475696 | 0.00069 | 0.147423 |
| XM_018048184.1 | HSP90B1 | 699.6743 | 765.1090 | -0.128981857 | 0.008506 | 0.284651 |
| XM_018048211.1 | CKAP4 | 166.1670 | 151.1126 | 0.137010527 | 0.029683 | 0.38551 |
| XM_018048221.1 | KPNA4 | 38.3701 | 31.4430 | 0.287242141 | 0.028187 | 0.381178 |
| XM_018048228.1 | SMC4 | 29.4841 | 21.9422 | 0.426227923 | 0.007468 | 0.268073 |
| XM_018048235.1 | TOM1 | 3.5584 | 5.1652 | -0.53761077 | 0.028997 | 0.383912 |
| XM_018048311.1 | LOC102180290 | 97.5903 | 10.1004 | 3.272330882 | 0.003413 | 0.196891 |
| XM_018048363.1 | TMTC1 | 8.8761 | 6.0361 | 0.556321123 | 0.002139 | 0.180463 |
| XM_018048373.1 | PPFIBP1 | 5.1757 | 4.8052 | 0.107175265 | 0.000146 | 0.083979 |
| XM_018048376.1 | ARNTL2 | 12.0786 | 9.3617 | 0.367607556 | 0.049393 | 0.448354 |
| XM_018048382.1 | SSPN | 6.9296 | 10.7830 | -0.637922607 | 0.026897 | 0.381178 |
| XM_018048413.1 | AEBP2 | 16.5348 | 13.6606 | 0.275488663 | 0.006524 | 0.25356 |
| XM_018048425.1 | MGST1 | 14.1377 | 16.6119 | -0.232673253 | 0.03032 | 0.388369 |
| XM_018048435.1 | EPS8 | 37.1898 | 21.2831 | 0.805194885 | 0.007026 | 0.261823 |
| XM_018048548.1 | TPI1 | 107.8531 | 112.5486 | -0.061480904 | 0.022227 | 0.351287 |
| XM_018048559.1 | MLF2 | 17.6168 | 21.9294 | -0.315914292 | 0.01812 | 0.33628 |
| XM_018048766.1 | DDX17 | 85.7651 | 101.7824 | -0.247024662 | 0.046439 | 0.440825 |
| XM_018048874.1 | TSPO | 75.8513 | 91.5128 | -0.270799329 | 0.048277 | 0.446267 |
| XM_018048880.1 | SAMM50 | 27.9896 | 30.4909 | -0.123486878 | 0.038415 | 0.416632 |
| XM_018048926.1 | LOC102184901 | 31.8631 | 9.6919 | 1.717038748 | 0.042821 | 0.430871 |
| XM_018048957.1 | MAPK12 | 7.9389 | 9.4687 | -0.2542394 | 0.004438 | 0.218418 |
| XM_018049221.1 | TIFA | 3.5544 | 1.9661 | 0.854219938 | 0.044873 | 0.437516 |
| XM_018049224.1 | C6H4orf32 | 11.8014 | 8.6638 | 0.445894776 | 0.013531 | 0.315345 |
| XM_018049271.1 | PPP3CA | 32.2743 | 28.2497 | 0.192148287 | 0.009541 | 0.295948 |
| XM_018049314.1 | HERC5 | 74.9088 | 10.4936 | 2.835626448 | 0.005606 | 0.242877 |
| XM_018049315.1 | HERC6 | 78.3115 | 7.7220 | 3.342181465 | 0.004574 | 0.22224 |
| XM_018049316.1 | HERC6 | 19.6613 | 3.3820 | 2.539427816 | 0.013999 | 0.315345 |
| XM_018049317.1 | PIGY | 47.8426 | 54.4291 | -0.18608321 | 0.049365 | 0.448354 |
| XM_018049578.1 | LOC102182395 | 19.7466 | 26.0070 | -0.397291639 | 0.020143 | 0.341604 |
| XM_018049717.1 | EVC | 6.3537 | 5.0934 | 0.318971864 | 0.046419 | 0.440825 |
| XM_018049727.1 | WDR1 | 87.3249 | 95.0048 | -0.121607069 | 0.01379 | 0.315345 |
| XM_018049826.1 | MXD4 | 8.9101 | 11.7900 | -0.404039072 | 0.019984 | 0.340825 |
| XM_018049968.1 | FKBP8 | 6.9805 | 9.6516 | -0.467424481 | 0.024659 | 0.369683 |
| XM_018050006.1 | PAM | 46.6008 | 41.4105 | 0.170358747 | 0.046095 | 0.440798 |
| XM_018050010.1 | CHD1 | 15.1663 | 15.2483 | -0.007781853 | 0.036196 | 0.410838 |
| XM_018050012.1 | LNPEP | 100.0619 | 65.2709 | 0.61638003 | 0.01692 | 0.332366 |
| XM_018050024.1 | RHOBTB3 | 205.0814 | 285.2368 | -0.475963505 | 0.028067 | 0.381178 |
| XM_018050076.1 | MAT2B | 29.9559 | 18.7819 | 0.673497266 | 0.010568 | 0.301764 |
| XM_018050128.1 | GALNT10 | 8.0055 | 4.7874 | 0.741750892 | 0.001396 | 0.173757 |
| XM_018050348.1 | TGFBI | 1683.9829 | 1865.1920 | -0.147446666 | 0.038709 | 0.41858 |
| XM_018050378.1 | JADE2 | 8.5807 | 4.8836 | 0.813161716 | 0.013836 | 0.315345 |
| XM_018050416.1 | MBD3 | 6.3550 | 7.8244 | -0.300085146 | 0.018672 | 0.336847 |
| XM_018050418.1 | MEX3D | 14.4311 | 16.3745 | -0.182264709 | 0.041947 | 0.42988 |
| XM_018050557.1 | MGAT1 | 30.6696 | 16.2864 | 0.913143573 | 0.028355 | 0.381178 |
| XM_018050655.1 | CLTB | 20.8439 | 24.5188 | -0.234266183 | 0.029714 | 0.38551 |
| XM_018050730.1 | LOC102189116 | 239.6960 | 256.6495 | -0.09859376 | 0.044057 | 0.435095 |
| XM_018050737.1 | KIF3A | 12.9402 | 11.4788 | 0.172893949 | 0.017335 | 0.334473 |
| XM_018050782.1 | SGTA | 4.9342 | 7.3997 | -0.584642757 | 0.012041 | 0.310278 |
| XM_018050850.1 | DAPK3 | 19.1312 | 23.8672 | -0.319104503 | 0.01521 | 0.321836 |
| XM_018050873.1 | UBXN6 | 2.5581 | 5.6272 | -1.137340202 | 0.01033 | 0.301266 |
| XM_018050913.1 | RPL36 | 28.6801 | 31.3910 | -0.130304648 | 0.011458 | 0.307914 |
| XM_018050998.1 | NDUFA7 | 19.8367 | 25.9469 | -0.387386209 | 0.027161 | 0.381178 |
| XM_018051015.1 | C7H19orf66 | 20.8160 | 5.9399 | 1.809182832 | 0.042703 | 0.430833 |
| XM_018051098.1 | SMARCA4 | 1.2064 | 2.4622 | -1.029206846 | 0.044826 | 0.437516 |
| XM_018051180.1 | PKN1 | 8.5431 | 10.6085 | -0.312400802 | 0.033241 | 0.398184 |
| XM_018051216.1 | C7H19orf53 | 20.5593 | 29.6643 | -0.528931731 | 0.004565 | 0.22224 |
| XM_018051239.1 | IER2 | 14.5341 | 18.0086 | -0.309239107 | 0.024012 | 0.364979 |
| XM_018051260.1 | PRDX2 | 117.7670 | 132.3923 | -0.168883828 | 0.027919 | 0.381178 |
| XM_018051273.1 | TNPO2 | 1.2448 | 2.6056 | -1.065747434 | 0.049496 | 0.448354 |
| XM_018051322.1 | DNAJC13 | 41.1850 | 27.4924 | 0.583086104 | 0.017102 | 0.333058 |
| XM_018051378.1 | DDA1 | 9.4458 | 9.6333 | -0.028356092 | 0.026815 | 0.381178 |
| XM_018051379.1 | BST2 | 31.7818 | 7.7253 | 2.04054474 | 0.038022 | 0.41402 |
| XM_018051380.1 | LOC108636404 | 97.3963 | 1.9430 | 5.647514873 | 0.000598 | 0.138845 |
| XM_018051386.1 | PGLS | 4.3334 | 6.2947 | -0.538627357 | 0.042317 | 0.430107 |
| XM_018051587.1 | ARF1 | 108.1158 | 119.0435 | -0.138912655 | 0.013612 | 0.315345 |
| XM_018051642.1 | MX2 | 145.9147 | 2.9417 | 5.632312557 | 0.001125 | 0.17009 |
| XM_018051644.1 | MX2 | 220.7060 | 0.0000 | Inf | 0.00027 | 0.111192 |
| XM_018051649.1 | MX1 | 704.3755 | 17.3595 | 5.342545397 | 8.78E-05 | 0.066252 |
| XM_018051653.1 | MX1 | 58.0696 | 2.1796 | 4.735619464 | 0.037926 | 0.41402 |
| XM_018051706.1 | LOC108636449 | 9.1682 | 0.1518 | 5.916430539 | 0.002943 | 0.193219 |
| XM_018051749.1 | CBS | 14.4016 | 15.0778 | -0.066202842 | 0.038803 | 0.41858 |
| XM_018052012.1 | RANBP6 | 7.9238 | 9.7419 | -0.298011848 | 0.003166 | 0.196891 |
| XM_018052050.1 | TJP2 | 8.0893 | 5.9343 | 0.446944903 | 0.036389 | 0.410838 |
| XM_018052139.1 | TPM2 | 224.0740 | 267.5490 | -0.255828399 | 0.019111 | 0.339544 |
| XM_018052198.1 | TDRD7 | 20.6856 | 5.6053 | 1.883760959 | 0.00824 | 0.27845 |
| XM_018052199.1 | TDRD7 | 3.6742 | 0.8738 | 2.072075767 | 0.037125 | 0.411631 |
| XM_018052352.1 | BNIP3L | 14.3639 | 18.2776 | -0.347627017 | 0.025089 | 0.372995 |
| XM_018052459.1 | GAS1 | 5.9755 | 0.1234 | 5.597829115 | 0.012629 | 0.312555 |
| XM_018052510.1 | NFIL3 | 42.3911 | 56.2762 | -0.408764223 | 0.003204 | 0.196891 |
| XM_018052545.1 | SLC44A1 | 173.3813 | 169.3955 | 0.033552668 | 0.020856 | 0.345787 |
| XM_018052777.1 | BAG1 | 18.6666 | 14.1266 | 0.402049646 | 0.035055 | 0.405676 |
| XM_018052872.1 | TMEM30A | 100.2526 | 123.9599 | -0.306234429 | 0.032883 | 0.3972 |
| XM_018052913.1 | FAM46A | 10.6817 | 2.5348 | 2.075205433 | 0.046076 | 0.440798 |
| XM_018052973.1 | EAF1 | 15.9004 | 13.7447 | 0.210180123 | 0.011849 | 0.309246 |
| XM_018053007.1 | MAN1A1 | 235.7320 | 199.9967 | 0.237171393 | 0.039934 | 0.422228 |
| XM_018053031.1 | RWDD1 | 34.6720 | 44.0669 | -0.345921391 | 0.046245 | 0.440825 |
| XM_018053035.1 | ANKRD28 | 94.5929 | 75.6426 | 0.322532314 | 0.01452 | 0.31684 |
| XM_018053041.1 | TSPYL4 | 6.0842 | 8.4984 | -0.482125403 | 0.018385 | 0.336847 |
| XM_018053045.1 | HDAC2 | 31.3665 | 33.9172 | -0.112791011 | 0.004348 | 0.218418 |
| XM_018053054.1 | FYN | 4.1801 | 5.9808 | -0.516803109 | 0.041345 | 0.427419 |
| XM_018053140.1 | GPR63 | 31.3677 | 15.5144 | 1.015672789 | 0.007956 | 0.276055 |
| XM_018053167.1 | SYNCRIP | 13.7733 | 10.9252 | 0.334219494 | 0.024038 | 0.364979 |
| XM_018053214.1 | EPB41L2 | 1.6424 | 2.7766 | -0.757534947 | 0.03099 | 0.391412 |
| XM_018053238.1 | KAT2B | 29.3811 | 26.0906 | 0.171359892 | 0.048497 | 0.446788 |
| XM_018053240.1 | AHI1 | 1.9734 | 0.0320 | 5.944377344 | 0.001838 | 0.180463 |
| XM_018053260.1 | HEBP2 | 46.1906 | 51.4654 | -0.15600241 | 0.04869 | 0.447199 |
| XM_018053357.1 | MTHFD1L | 15.6615 | 13.1681 | 0.250169938 | 0.007548 | 0.268073 |
| XM_018053416.1 | TMEM181 | 19.2568 | 22.4408 | -0.22075279 | 0.017384 | 0.334473 |
| XM_018053456.1 | LOC108636750 | 6.0687 | 3.6619 | 0.728799029 | 0.013847 | 0.315345 |
| XM_018053459.1 | MPC1 | 14.1637 | 17.0514 | -0.267685362 | 0.025537 | 0.374212 |
| XM_018053492.1 | C9H6orf120 | 9.7521 | 13.6727 | -0.487517857 | 0.019226 | 0.339544 |
| XM_018053661.1 | SPG21 | 8.6593 | 11.5969 | -0.42142642 | 0.03885 | 0.41858 |
| XM_018053703.1 | HMGCR | 24.2304 | 22.1748 | 0.127894226 | 0.049277 | 0.448354 |
| XM_018054041.1 | GALNT16 | 16.2485 | 21.8192 | -0.425296342 | 0.008933 | 0.289542 |
| XM_018054116.1 | WDHD1 | 12.7412 | 10.0358 | 0.34434662 | 0.003647 | 0.204863 |
| XM_018054118.1 | GBE1 | 40.6488 | 43.5782 | -0.100395059 | 0.009563 | 0.295948 |
| XM_018054170.1 | DUT | 16.5456 | 20.2556 | -0.291870664 | 0.033554 | 0.400355 |
| XM_018054177.1 | DTWD1 | 1.3970 | 2.3141 | -0.728057424 | 0.042075 | 0.42988 |
| XM_018054201.1 | TMOD2 | 8.4647 | 6.1728 | 0.455544657 | 0.002405 | 0.186186 |
| XM_018054203.1 | TMOD3 | 33.4155 | 31.2012 | 0.098916902 | 0.036786 | 0.411631 |
| XM_018054271.1 | ADAM10 | 55.1070 | 52.6816 | 0.064935606 | 0.043508 | 0.433451 |
| XM_018054296.1 | TPM1 | 10.7333 | 18.5764 | -0.791377461 | 0.030111 | 0.387339 |
| XM_018054299.1 | TPM1 | 26.4497 | 45.6272 | -0.786644354 | 0.010596 | 0.301764 |
| XM_018054305.1 | TPM1 | 2.0533 | 4.2187 | -1.038885208 | 0.044571 | 0.436832 |
| XM_018054306.1 | TPM1 | 1.8041 | 2.6995 | -0.581466108 | 0.04775 | 0.445351 |
| XM_018054307.1 | TPM1 | 2.0408 | 3.4862 | -0.772534298 | 0.04576 | 0.439455 |
| XM_018054311.1 | LACTB | 15.3677 | 20.3890 | -0.407892791 | 0.015042 | 0.321836 |
| XM_018054439.1 | GCHFR | 6.7191 | 8.5665 | -0.350433271 | 0.043265 | 0.43296 |
| XM_018054498.1 | GREM1 | 48.5472 | 44.5903 | 0.122658256 | 0.000872 | 0.151903 |
| XM_018054510.1 | PARP2 | 5.9130 | 7.6794 | -0.377104092 | 0.000781 | 0.147423 |
| XM_018054592.1 | HOMEZ | 7.5552 | 9.2215 | -0.287526128 | 0.037272 | 0.411631 |
| XM_018054684.1 | NECAP2 | 6.5556 | 8.6783 | -0.40468873 | 0.004964 | 0.234012 |
| XM_018054751.1 | DPH7 | 12.8931 | 13.9306 | -0.111650393 | 0.027231 | 0.381178 |
| XM_018054752.1 | NELFB | 7.3855 | 8.9463 | -0.276590228 | 0.002131 | 0.180463 |
| XM_018054770.1 | EMC1 | 9.7401 | 11.6378 | -0.256815859 | 0.018043 | 0.33628 |
| XM_018054825.1 | MRPS5 | 18.7875 | 20.9464 | -0.156928713 | 0.030436 | 0.388754 |
| XM_018055121.1 | EIF2AK2 | 164.1955 | 25.5056 | 2.686527245 | 0.000948 | 0.154796 |
| XM_018055123.1 | EIF2AK2 | 38.8467 | 5.0296 | 2.949283673 | 0.002229 | 0.180463 |
| XM_018055131.1 | CDC42EP3 | 61.8975 | 83.6691 | -0.434814653 | 0.016527 | 0.331741 |
| XM_018055202.1 | KCNK12 | 37.5751 | 28.0749 | 0.420495581 | 0.025151 | 0.372995 |
| XM_018055203.1 | MSH6 | 9.0765 | 21.8169 | -1.265236804 | 0.034467 | 0.402754 |
| XM_018055324.1 | USP48 | 4.8399 | 5.0226 | -0.05345109 | 0.047363 | 0.443552 |
| XM_018055330.1 | USP48 | 4.4650 | 4.3448 | 0.039364867 | 0.022652 | 0.356348 |
| XM_018055354.1 | RMND5A | 17.9086 | 19.7477 | -0.141032689 | 0.00075 | 0.147423 |
| XM_018055380.1 | MAT2A | 63.4352 | 45.9564 | 0.465017511 | 0.017954 | 0.335928 |
| XM_018055390.1 | SUCLG1 | 46.1176 | 49.0274 | -0.088270159 | 0.048558 | 0.446888 |
| XM_018055493.1 | MRPL33 | 6.7327 | 10.9812 | -0.705772891 | 0.033962 | 0.401488 |
| XM_018055661.1 | ODC1 | 198.2858 | 146.6756 | 0.434952507 | 0.031435 | 0.393455 |
| XM_018055667.1 | CYS1 | 5.1626 | 7.1784 | -0.475566082 | 0.00254 | 0.188329 |
| XM_018055702.1 | RSAD2 | 195.6947 | 0.4317 | 8.824195926 | 0.0012 | 0.17009 |
| XM_018055703.1 | CMPK2 | 95.2818 | 2.1563 | 5.465545669 | 0.001484 | 0.173757 |
| XM_018056078.1 | UBAC1 | 9.4014 | 10.8478 | -0.206458097 | 0.000495 | 0.137526 |
| XM_018056130.1 | AGPAT2 | 13.8201 | 10.9188 | 0.339944798 | 0.04924 | 0.448354 |
| XM_018056283.1 | TNFSF13B | 3.1854 | 0.0296 | 6.749800813 | 0.049678 | 0.448354 |
| XM_018056622.1 | B3GLCT | 12.0959 | 15.6242 | -0.369265756 | 0.016054 | 0.331741 |
| XM_018056673.1 | SUPT20H | 3.9761 | 1.9699 | 1.013231635 | 0.028401 | 0.381178 |
| XM_018056782.1 | EPSTI1 | 39.8321 | 0.9064 | 5.457678119 | 0.001434 | 0.173757 |
| XM_018056822.1 | DIAPH3 | 6.7777 | 4.5819 | 0.564864208 | 0.043488 | 0.433451 |
| XM_018056827.1 | IFI6 | 546.0067 | 115.9468 | 2.235455207 | 0.008152 | 0.276602 |
| XM_018056841.1 | FAM76A | 11.8345 | 7.9533 | 0.573373219 | 0.020093 | 0.341604 |
| XM_018056866.1 | DUSP15 | 4.1227 | 0.7241 | 2.509356081 | 0.010379 | 0.301266 |
| XM_018056868.1 | MRPS6 | 25.0220 | 24.9948 | 0.001566838 | 0.013604 | 0.315345 |
| XM_018056925.1 | BTBD3 | 5.9312 | 3.2416 | 0.871602285 | 0.000426 | 0.133828 |
| XM_018056926.1 | BTBD3 | 15.9592 | 11.6870 | 0.449482287 | 0.029244 | 0.3854 |
| XM_018057012.1 | CAMK1D | 5.8169 | 3.9524 | 0.557519666 | 0.002789 | 0.18989 |
| XM_018057017.1 | LOC102175492 | 1.8864 | 0.1154 | 4.03054668 | 0.004033 | 0.208709 |
| XM_018057058.1 | MINA | 6.4401 | 4.7445 | 0.440812759 | 0.04531 | 0.437516 |
| XM_018057150.1 | FAM188A | 13.0457 | 1.1316 | 3.527084795 | 0.006101 | 0.25356 |
| XM_018057180.1 | ZEB1 | 4.1220 | 1.7402 | 1.244055751 | 0.006445 | 0.25356 |
| XM_018057268.1 | XRN2 | 83.3376 | 28.5118 | 1.547407984 | 0.003373 | 0.196891 |
| XM_018057322.1 | CLDND1 | 29.8586 | 22.9404 | 0.380255233 | 0.009446 | 0.294985 |
| XM_018057376.1 | FERMT1 | 6.7428 | 4.8287 | 0.481700786 | 0.035203 | 0.406117 |
| XM_018057460.1 | GINS1 | 14.6512 | 9.6997 | 0.595009388 | 0.026224 | 0.377377 |
| XM_018057534.1 | PPDPF | 10.3164 | 16.6168 | -0.68770456 | 0.033053 | 0.397516 |
| XM_018057577.1 | OGFR | 35.6297 | 12.9232 | 1.463115842 | 0.042007 | 0.42988 |
| XM_018057587.1 | ADRM1 | 12.8013 | 13.8266 | -0.111159912 | 0.014486 | 0.31684 |
| XM_018057588.1 | OSBPL2 | 20.2735 | 23.2614 | -0.198340623 | 0.018737 | 0.337338 |
| XM_018057637.1 | FAM210B | 50.9592 | 62.2620 | -0.289007835 | 0.009361 | 0.293554 |
| XM_018057642.1 | FKBP1A | 80.0796 | 82.0905 | -0.035779879 | 0.025528 | 0.374212 |
| XM_018057661.1 | CSNK2A1 | 41.7550 | 47.2380 | -0.178001236 | 0.043282 | 0.43296 |
| XM_018057731.1 | RALY | 9.5353 | 11.4541 | -0.264520937 | 0.012587 | 0.312555 |
| XM_018057739.1 | MAP1LC3A | 12.4342 | 18.4610 | -0.570164917 | 0.016794 | 0.332248 |
| XM_018057740.1 | MAP1LC3A | 6.5297 | 9.7804 | -0.582885499 | 0.014278 | 0.31684 |
| XM_018057813.1 | MYL9 | 34.8225 | 54.2831 | -0.640485426 | 0.038402 | 0.416632 |
| XM_018057820.1 | ST3GAL6 | 4.6580 | 1.9570 | 1.251020255 | 0.046661 | 0.441549 |
| XM_018057897.1 | JPH2 | 9.2869 | 13.9996 | -0.592127037 | 0.012581 | 0.312555 |
| XM_018057962.1 | ELMO2 | 10.3590 | 7.7287 | 0.422582796 | 0.00124 | 0.17009 |
| XM_018058006.1 | ZNFX1 | 51.4165 | 12.7800 | 2.008346786 | 0.00114 | 0.17009 |
| XM_018058014.1 | RNF114 | 22.9794 | 8.9941 | 1.353287229 | 0.007179 | 0.264494 |
| XM_018058029.1 | BCAS4 | 14.6528 | 18.8737 | -0.36519544 | 0.01599 | 0.331362 |
| XM_018058050.1 | FNDC5 | 6.4424 | 8.8611 | -0.459890204 | 0.016997 | 0.332366 |
| XM_018058064.1 | TRAM1 | 47.3329 | 40.5986 | 0.221413362 | 0.029514 | 0.3854 |
| XM_018058065.1 | EEF1D | 1.0597 | 2.0667 | -0.96368365 | 0.022502 | 0.355009 |
| XM_018058184.1 | CPNE3 | 63.4958 | 71.7242 | -0.175798353 | 0.010104 | 0.301266 |
| XM_018058211.1 | FAM92A1 | 3.9661 | 8.2732 | -1.060731935 | 0.003334 | 0.196891 |
| XM_018058212.1 | RBM12B | 4.0728 | 2.3403 | 0.799335115 | 0.026662 | 0.380342 |
| XM_018058250.1 | GDF6 | 14.3166 | 8.4740 | 0.756576955 | 0.002024 | 0.180463 |
| XM_018058253.1 | PTDSS1 | 27.1038 | 29.9315 | -0.143171616 | 0.027814 | 0.381178 |
| XM_018058281.1 | STK3 | 13.2316 | 14.4251 | -0.124597803 | 0.014145 | 0.31684 |
| XM_018058353.1 | EBAG9 | 0.0729 | 3.4705 | -5.573947306 | 0.000787 | 0.147423 |
| XM_018058398.1 | TMEM70 | 32.0398 | 5.4326 | 2.560161993 | 0.001893 | 0.180463 |
| XM_018058399.1 | TMEM70 | 2.0437 | 24.8363 | -3.60322787 | 0.001655 | 0.180463 |
| XM_018058474.1 | ASPH | 3.4046 | 2.1105 | 0.68987795 | 0.032401 | 0.395454 |
| XM_018058477.1 | ASPH | 9.2415 | 6.9532 | 0.410448538 | 0.012092 | 0.310278 |
| XM_018058514.1 | RB1CC1 | 37.3229 | 34.8262 | 0.099885975 | 0.046094 | 0.440798 |
| XM_018058529.1 | CEBPD | 103.1513 | 123.8597 | -0.263945064 | 0.014255 | 0.31684 |
| XM_018058536.1 | ATAD2 | 21.6056 | 15.6166 | 0.468318677 | 0.038945 | 0.41858 |
| XM_018058575.1 | EFR3A | 6.6360 | 3.8915 | 0.769995384 | 0.045597 | 0.438814 |
| XM_018058589.1 | NDRG1 | 36.0508 | 30.4782 | 0.242257353 | 0.032815 | 0.3972 |
| XM_018058614.1 | AGO2 | 12.3654 | 9.1499 | 0.434477346 | 0.029386 | 0.3854 |
| XM_018058697.1 | PARP10 | 16.5210 | 5.1423 | 1.683805722 | 0.013944 | 0.315345 |
| XM_018058698.1 | GRINA | 11.8711 | 7.1330 | 0.734877568 | 0.029287 | 0.3854 |
| XM_018058746.1 | VPS28 | 8.3403 | 11.8197 | -0.503016625 | 0.006421 | 0.25356 |
| XM_018058828.1 | OGFRL1 | 12.7400 | 16.3049 | -0.355937752 | 0.030631 | 0.389594 |
| XM_018058933.1 | LOC102191717 | 44.9465 | 18.2812 | 1.297844803 | 0.039818 | 0.422228 |
| XM_018059058.1 | UBE2L6 | 75.3407 | 18.4721 | 2.028081683 | 0.025239 | 0.372995 |
| XM_018059065.1 | TIMM10 | 11.8627 | 7.3149 | 0.69751697 | 0.036269 | 0.410838 |
| XM_018059089.1 | NDUFS3 | 16.8395 | 18.5927 | -0.142889207 | 0.028412 | 0.381178 |
| XM_018059123.1 | PACSIN3 | 6.6841 | 8.6779 | -0.376621866 | 0.009674 | 0.297988 |
| XM_018059282.1 | QSER1 | 8.9617 | 5.0658 | 0.822984001 | 0.019534 | 0.339544 |
| XM_018059305.1 | LGR4 | 13.1558 | 9.5131 | 0.467716725 | 0.01311 | 0.312555 |
| XM_018059404.1 | THYN1 | 12.0435 | 13.8243 | -0.19894841 | 0.027581 | 0.381178 |
| XM_018059416.1 | NUMA1 | 7.3061 | 9.8597 | -0.432436896 | 0.000798 | 0.147423 |
| XM_018059444.1 | LOC102169889 | 76.7786 | 6.5809 | 3.544346213 | 0.007185 | 0.264494 |
| XM_018059445.1 | LOC108637671 | 3.6060 | 0.2775 | 3.699838514 | 0.029565 | 0.3854 |
| XM_018059469.1 | ARFIP2 | 13.0752 | 15.3265 | -0.229191852 | 0.014646 | 0.31789 |
| XM_018059472.1 | ILK | 52.2172 | 59.4871 | -0.188049698 | 0.034698 | 0.404406 |
| XM_018059479.1 | LOC108633278 | 16.2131 | 4.4260 | 1.873081885 | 0.041071 | 0.426138 |
| XM_018059520.1 | DENND5A | 45.5573 | 38.2009 | 0.254075502 | 0.000432 | 0.133828 |
| XM_018059548.1 | USP47 | 3.6875 | 2.4651 | 0.581028692 | 0.038958 | 0.41858 |
| XM_018059587.1 | ARPC2 | 152.4376 | 154.3078 | -0.017592556 | 0.009943 | 0.301266 |
| XM_018059684.1 | LOC108637680 | 13.5618 | 11.1925 | 0.277021524 | 0.009029 | 0.289542 |
| XM_018059729.1 | FXYD6 | 11.4500 | 14.2265 | -0.3132326 | 0.034649 | 0.404355 |
| XM_018059745.1 | SIK3 | 3.1362 | 2.5385 | 0.305073183 | 0.01843 | 0.336847 |
| XM_018059843.1 | SLC35F2 | 6.5215 | 2.5909 | 1.33175131 | 0.031082 | 0.392033 |
| XM_018059862.1 | ENDOD1 | 18.7713 | 13.7834 | 0.445597136 | 0.019341 | 0.339544 |
| XM_018060017.1 | MDM4 | 18.3281 | 13.1725 | 0.476536116 | 0.037124 | 0.411631 |
| XM_018060148.1 | TROVE2 | 7.9508 | 10.0817 | -0.342561291 | 0.006428 | 0.25356 |
| XM_018060165.1 | BPNT1 | 16.4695 | 20.0020 | -0.280347292 | 0.021422 | 0.348286 |
| XM_018060188.1 | AIDA | 119.2931 | 70.9001 | 0.750650215 | 0.013153 | 0.312638 |
| XM_018060204.1 | DEGS1 | 101.3112 | 81.5712 | 0.312661781 | 0.04642 | 0.440825 |
| XM_018060253.1 | DESI2 | 14.0333 | 18.9295 | -0.431786404 | 0.037623 | 0.413911 |
| XM_018060261.1 | AKT3 | 39.9472 | 38.5955 | 0.049661148 | 0.010989 | 0.302822 |
| XM_018060271.1 | CEP170 | 4.1411 | 2.4988 | 0.728761359 | 0.043313 | 0.43296 |
| XM_018060294.1 | PRRX1 | 15.6530 | 20.2309 | -0.370120762 | 0.012247 | 0.310879 |
| XM_018060363.1 | KIF1B | 2.4005 | 3.0227 | -0.332490985 | 0.00371 | 0.204863 |
| XM_018060414.1 | THAP3 | 4.8692 | 6.0437 | -0.311741224 | 0.024039 | 0.364979 |
| XM_018060445.1 | ICMT | 8.2165 | 13.7175 | -0.739415724 | 0.048822 | 0.447956 |
| XM_018060458.1 | C16H1orf174 | 15.2166 | 16.9713 | -0.157453544 | 0.049807 | 0.448354 |
| XM_018060465.1 | TPRG1L | 43.6894 | 47.4109 | -0.117936554 | 0.001472 | 0.173757 |
| XM_018060542.1 | CPTP | 11.1219 | 15.0814 | -0.439377302 | 0.004987 | 0.234012 |
| XM_018060561.1 | AGRN | 7.3456 | 3.0685 | 1.259378801 | 0.020574 | 0.345612 |
| XM_018060588.1 | PLEKHM2 | 10.1496 | 9.3580 | 0.117154043 | 0.014515 | 0.31684 |
| XM_018060688.1 | RNASEL | 3.1792 | 1.1597 | 1.454936968 | 0.0186 | 0.336847 |
| XM_018060759.1 | LPGAT1 | 29.0525 | 11.5909 | 1.325665978 | 0.002976 | 0.193219 |
| XM_018060879.1 | CSRP1 | 45.6866 | 56.2554 | -0.300221047 | 0.014776 | 0.318598 |
| XM_018060881.1 | CSRP1 | 1.5120 | 3.8319 | -1.341606078 | 0.013531 | 0.315345 |
| XM_018060882.1 | PHLDA3 | 25.6180 | 30.7917 | -0.265383941 | 0.037766 | 0.413911 |
| XM_018060897.1 | MIF | 93.2494 | 102.0429 | -0.130010209 | 0.009269 | 0.293497 |
| XM_018060921.1 | YWHAH | 46.4691 | 51.3665 | -0.144554181 | 0.019196 | 0.339544 |
| XM_018061093.1 | UNG | 24.0655 | 17.8172 | 0.433696079 | 0.02436 | 0.367671 |
| XM_018061128.1 | DYNLL1 | 114.0356 | 133.7919 | -0.230505733 | 0.00059 | 0.138845 |
| XM_018061129.1 | SRSF9 | 30.8643 | 29.8089 | 0.050198039 | 0.033019 | 0.397516 |
| XM_018061148.1 | NAA25 | 14.5168 | 12.2864 | 0.240656156 | 0.049882 | 0.448354 |
| XM_018061161.1 | LOC102190983 | 55.6444 | 0.0573 | 9.922956923 | 1.54E-06 | 0.013964 |
| XM_018061162.1 | LOC102190983 | 376.7097 | 4.0347 | 6.544832176 | 9.93E-06 | 0.040045 |
| XM_018061163.1 | LOC102186893 | 30.1467 | 0.0529 | 9.154834189 | 0.000444 | 0.133828 |
| XM_018061164.1 | LOC108637852 | 4.4660 | 0.0412 | 6.761097546 | 0.003443 | 0.197336 |
| XM_018061200.1 | SPATS2L | 10.8391 | 0.8021 | 3.75630499 | 0.016643 | 0.331988 |
| XM_018061305.1 | DIABLO | 21.9161 | 24.1498 | -0.140021495 | 0.006055 | 0.25356 |
| XM_018061374.1 | ZNF664 | 1.2531 | 1.4673 | -0.227662806 | 0.049356 | 0.448354 |
| XM_018061466.1 | ZNF605 | 0.3279 | 1.0540 | -1.68454455 | 0.013408 | 0.315345 |
| XM_018061492.1 | RAPGEF2 | 2.0430 | 3.0592 | -0.582446119 | 0.013435 | 0.315345 |
| XM_018061493.1 | RAPGEF2 | 2.0431 | 1.9694 | 0.052995411 | 0.047804 | 0.445351 |
| XM_018061520.1 | MYO1B | 12.8087 | 11.4006 | 0.168013106 | 0.049815 | 0.448354 |
| XM_018061528.1 | JADE1 | 3.1782 | 0.9701 | 1.712002564 | 0.034397 | 0.402471 |
| XM_018061553.1 | STAT1 | 239.9410 | 61.0386 | 1.974885838 | 0.004696 | 0.226117 |
| XM_018061571.1 | OTUD4 | 16.3237 | 10.2310 | 0.674018482 | 0.012768 | 0.312555 |
| XM_018061590.1 | LRBA | 25.1496 | 21.4026 | 0.232752295 | 0.025115 | 0.372995 |
| XM_018061890.1 | SPG7 | 6.5628 | 7.9726 | -0.280735686 | 0.009368 | 0.293554 |
| XM_018061918.1 | CHMP1A | 13.4241 | 16.4962 | -0.297302222 | 0.000931 | 0.154796 |
| XM_018061957.1 | N4BP1 | 17.1230 | 7.6724 | 1.158189369 | 0.003872 | 0.204863 |
| XM_018061990.1 | AKTIP | 8.7723 | 11.8539 | -0.434338376 | 0.034877 | 0.404931 |
| XM_018062097.1 | CES2 | 12.7053 | 15.4043 | -0.277901487 | 0.018655 | 0.336847 |
| XM_018062113.1 | NOL3 | 12.0885 | 14.6585 | -0.278104462 | 0.047479 | 0.443964 |
| XM_018062213.1 | NFAT5 | 0.9862 | 4.2639 | -2.112246112 | 0.025712 | 0.374829 |
| XM_018062223.1 | IST1 | 2.3066 | 18.5941 | -3.010991456 | 0.047352 | 0.443552 |
| XM_018062257.1 | DPY19L3 | 18.5195 | 28.5781 | -0.625866704 | 0.008664 | 0.285314 |
| XM_018062263.1 | R3HDM1 | 7.8020 | 6.0297 | 0.371748144 | 0.003744 | 0.204863 |
| XM_018062388.1 | SAMD4B | 0.5907 | 2.0524 | -1.796765219 | 0.006217 | 0.25356 |
| XM_018062561.1 | APOE | 6.4876 | 7.3579 | -0.181603924 | 0.012968 | 0.312555 |
| XM_018062621.1 | QPCTL | 6.5403 | 9.1766 | -0.488596486 | 0.016572 | 0.331741 |
| XM_018062643.1 | CALM3 | 10.9719 | 17.0909 | -0.639412552 | 0.019379 | 0.339544 |
| XM_018062749.1 | NUCB1 | 56.4900 | 69.7378 | -0.30394649 | 0.044912 | 0.437516 |
| XM_018062774.1 | NMI | 30.2786 | 14.7094 | 1.04155759 | 0.036523 | 0.410919 |
| XM_018062789.1 | FCGRT | 44.9974 | 51.6312 | -0.198403786 | 0.031644 | 0.393623 |
| XM_018062821.1 | PTOV1 | 17.4484 | 21.0702 | -0.272102725 | 0.012158 | 0.310278 |
| XM_018063239.1 | CD47 | 98.8931 | 63.0991 | 0.648251081 | 0.018637 | 0.336847 |
| XM_018063420.1 | WDSUB1 | 1.4456 | 2.5398 | -0.813060576 | 0.027982 | 0.381178 |
| XM_018063480.1 | TSEN34 | 11.8453 | 13.4144 | -0.179469737 | 0.020608 | 0.345612 |
| XM_018063554.1 | CHMP2A | 40.2305 | 44.0485 | -0.130803342 | 0.005136 | 0.235447 |
| XM_018063577.1 | MCRIP1 | 2.6003 | 7.1244 | -1.454110429 | 0.043609 | 0.433856 |
| XM_018063686.1 | TK1 | 12.7855 | 14.9542 | -0.22604263 | 0.020679 | 0.345787 |
| XM_018063731.1 | MXRA7 | 9.2101 | 11.5273 | -0.323761753 | 0.005869 | 0.25064 |
| XM_018063733.1 | MXRA7 | 28.1135 | 35.9178 | -0.353435876 | 0.002987 | 0.193219 |
| XM_018063804.1 | GALK1 | 10.2213 | 10.9519 | -0.099604018 | 0.000142 | 0.083979 |
| XM_018063834.1 | IFIH1 | 105.4451 | 8.5631 | 3.62221008 | 0.000765 | 0.147423 |
| XM_018063905.1 | SOX9 | 5.1536 | 2.7345 | 0.91431155 | 0.0078 | 0.273581 |
| XM_018063944.1 | AMZ2 | 5.5771 | 7.0335 | -0.334734607 | 0.034328 | 0.402471 |
| XM_018064164.1 | DHRS11 | 6.6185 | 8.2696 | -0.321321678 | 0.029577 | 0.3854 |
| XM_018064240.1 | TMEM98 | 21.6047 | 26.2112 | -0.278841513 | 0.041421 | 0.427716 |
| XM_018064263.1 | CRLF3 | 20.6588 | 25.9122 | -0.326877282 | 0.005457 | 0.242877 |
| XM_018064270.1 | NF1 | 1.8626 | 5.1147 | -1.457356797 | 0.014012 | 0.315345 |
| XM_018064360.1 | BLMH | 18.1277 | 19.6713 | -0.117899626 | 0.012706 | 0.312555 |
| XM_018064367.1 | NXN | 6.7222 | 9.6850 | -0.526807485 | 0.018299 | 0.336847 |
| XM_018064493.1 | KIF1C | 14.7611 | 18.8061 | -0.349401532 | 0.030863 | 0.3909 |
| XM_018064540.1 | PFN1 | 179.2002 | 197.9281 | -0.143404167 | 0.024716 | 0.369683 |
| XM_018064553.1 | ACADVL | 0.0000 | 2.6161 | #NAME? | 3.02E-05 | 0.040045 |
| XM_018064564.1 | LOC100861136 | 33.1966 | 37.8378 | -0.188790705 | 0.003366 | 0.196891 |
| XM_018064566.1 | GPS2 | 6.6360 | 8.6271 | -0.378561458 | 0.022081 | 0.351287 |
| XM_018064636.1 | MYH10 | 12.6197 | 15.9638 | -0.339128243 | 0.00681 | 0.257299 |
| XM_018064673.1 | ITGA6 | 6.6345 | 3.7499 | 0.823123738 | 0.000957 | 0.154796 |
| XM_018064676.1 | ITGA6 | 89.9152 | 58.3102 | 0.624817203 | 0.013835 | 0.315345 |
| XM_018064684.1 | PDK1 | 3.5147 | 6.4308 | -0.871592266 | 0.007586 | 0.268359 |
| XM_018064778.1 | CDCA7 | 5.8386 | 3.9879 | 0.549982681 | 0.000458 | 0.133828 |
| XM_018064917.1 | ZNF652 | 3.9040 | 1.6779 | 1.218284317 | 0.021615 | 0.349258 |
| XM_018064944.1 | NFE2L1 | 11.6096 | 12.8250 | -0.143642439 | 0.01334 | 0.315345 |
| XM_018064945.1 | NFE2L1 | 10.5062 | 13.2808 | -0.33810068 | 0.037561 | 0.413911 |
| XM_018064948.1 | NFE2L1 | 11.4897 | 15.2158 | -0.405228413 | 0.002558 | 0.188329 |
| XM_018064951.1 | COPZ2 | 17.2494 | 26.2848 | -0.607682287 | 0.019414 | 0.339544 |
| XM_018065038.1 | SMARCE1 | 11.2463 | 12.8288 | -0.189946837 | 0.008765 | 0.287607 |
| XM_018065040.1 | MTX2 | 0.7290 | 4.0764 | -2.483287883 | 0.045181 | 0.437516 |
| XM_018065101.1 | CNP | 19.9288 | 7.0601 | 1.497091841 | 0.018121 | 0.33628 |
| XM_018065104.1 | DHX58 | 13.2783 | 1.2929 | 3.36037052 | 0.004071 | 0.209479 |
| XM_018065106.1 | DHX58 | 16.2756 | 3.3364 | 2.286326843 | 0.027549 | 0.381178 |
| XM_018065137.1 | TUBG1 | 6.5375 | 7.3460 | -0.16822081 | 0.00926 | 0.293497 |
| XM_018065161.1 | VAT1 | 51.4679 | 68.3116 | -0.408459358 | 0.005619 | 0.242877 |
| XM_018065166.1 | BRCA1 | 9.1451 | 7.2351 | 0.337981473 | 0.032971 | 0.397516 |
| XM_018065362.1 | SMURF2 | 74.1993 | 57.3343 | 0.37200709 | 0.024061 | 0.364979 |
| XM_018065422.1 | DNAJC10 | 38.1982 | 46.6489 | -0.288338256 | 0.021787 | 0.350449 |
| XM_018065470.1 | FAM171B | 47.6928 | 33.9452 | 0.490563591 | 0.003914 | 0.204863 |
| XM_018065497.1 | DHX29 | 23.1469 | 21.0843 | 0.134649791 | 0.036761 | 0.411631 |
| XM_018065588.1 | RAI14 | 5.7767 | 6.7374 | -0.22193551 | 0.044172 | 0.435278 |
| XM_018065618.1 | RICTOR | 11.8438 | 7.7928 | 0.603925492 | 0.019293 | 0.339544 |
| XM_018065635.1 | OSMR | 26.9971 | 20.3083 | 0.410735514 | 0.013878 | 0.315345 |
| XM_018065656.1 | SDHA | 53.5161 | 59.0916 | -0.142979584 | 0.002488 | 0.188329 |
| XM_018065673.1 | NADK2 | 0.1674 | 2.5418 | -3.9241621 | 0.002644 | 0.189392 |
| XM_018065841.1 | LIMS2 | 3.1412 | 5.1603 | -0.716131398 | 0.036328 | 0.410838 |
| XM_018065866.1 | NUDT14 | 7.2918 | 8.2005 | -0.169431716 | 0.03095 | 0.391412 |
| XM_018065999.1 | FAM177A1 | 11.7246 | 13.3787 | -0.190398694 | 0.02156 | 0.349258 |
| XM_018066021.1 | IDH2 | 11.9551 | 13.8962 | -0.217068995 | 0.00714 | 0.264494 |
| XM_018066108.1 | FES | 7.3750 | 6.4298 | 0.197871904 | 0.032484 | 0.395921 |
| XM_018066160.1 | LOC106503930 | 8.5774 | 0.1244 | 6.10751876 | 0.010371 | 0.301266 |
| XM_018066226.1 | PRPF39 | 2.1258 | 7.9856 | -1.909416424 | 0.009864 | 0.301266 |
| XM_018066296.1 | CEMIP | 116.2831 | 255.7411 | -1.137043459 | 0.026797 | 0.381178 |
| XM_018066347.1 | SEC23A | 158.0548 | 192.4955 | -0.284400202 | 0.007484 | 0.268073 |
| XM_018066491.1 | LOC102172846 | 7.9496 | 10.7427 | -0.43439439 | 0.038965 | 0.41858 |
| XM_018066492.1 | EAPP | 15.3153 | 16.7193 | -0.126534183 | 0.014479 | 0.31684 |
| XM_018066538.1 | LYSMD4 | 2.0350 | 1.6372 | 0.313802993 | 0.042565 | 0.430833 |
| XM_018066656.1 | APEH | 4.6805 | 5.8166 | -0.313515627 | 0.018616 | 0.336847 |
| XM_018066711.1 | PBRM1 | 2.6200 | 3.3358 | -0.348431649 | 0.025357 | 0.373992 |
| XM_018066910.1 | FAM198A | 1.9215 | 3.9848 | -1.052297033 | 0.043125 | 0.43296 |
| XM_018066918.1 | PCBP4 | 7.9653 | 11.8968 | -0.578764385 | 0.031569 | 0.393455 |
| XM_018066924.1 | TADA3 | 2.0863 | 2.4813 | -0.250152777 | 0.018386 | 0.336847 |
| XM_018066929.1 | ARPC4 | 47.5425 | 53.3440 | -0.166106285 | 0.039989 | 0.422228 |
| XM_018066931.1 | PTPRG | 1.6399 | 0.8866 | 0.887289222 | 0.027885 | 0.381178 |
| XM_018067010.1 | UBP1 | 8.1863 | 6.6618 | 0.297289001 | 0.031536 | 0.393455 |
| XM_018067025.1 | TREX1 | 10.4247 | 2.7581 | 1.918278604 | 0.00851 | 0.284651 |
| XM_018067028.1 | SHISA5 | 22.1726 | 7.3974 | 1.583685286 | 0.003816 | 0.204863 |
| XM_018067029.1 | AZI2 | 22.5014 | 4.4881 | 2.325827747 | 0.01004 | 0.301266 |
| XM_018067049.1 | TKT | 44.7469 | 62.0834 | -0.472418848 | 0.048954 | 0.44826 |
| XM_018067182.1 | TRANK1 | 13.4763 | 0.8272 | 4.026094654 | 4.16E-05 | 0.047118 |
| XR_001917668.1 | LOC102173185 | 2.1453 | 0.3339 | 2.683515596 | 0.034869 | 0.404931 |
| XR_001917825.1 | IFI44L | 22.3814 | 0.6819 | 5.03649855 | 0.008125 | 0.276602 |
| XR_001917826.1 | IFI44 | 4.8194 | 0.2208 | 4.448185338 | 0.016542 | 0.331741 |
| XR_001918781.1 | SRSF7 | 2.4689 | 1.7985 | 0.457040399 | 0.022704 | 0.356348 |
| XR_001919025.1 | IDH3B | 3.3577 | 3.6545 | -0.12217679 | 0.045391 | 0.437574 |
| XR_001919541.1 | IRF3 | 2.9084 | 1.1637 | 1.321537016 | 0.029448 | 0.3854 |
| XR_001919647.1 | CEP112 | 3.3944 | 1.2965 | 1.3885015 | 0.025499 | 0.374212 |
| XR_001919788.1 | ASNSD1 | 6.6418 | 0.5782 | 3.521979265 | 0.003313 | 0.196891 |
